# *Babesia microti* Confers Macrophage-Based Cross-Protective Immunity Against Murine Malaria

**DOI:** 10.3389/fcimb.2020.00193

**Published:** 2020-04-29

**Authors:** Artemis Efstratiou, Eloiza May S. Galon, Guanbo Wang, Kousuke Umeda, Daisuke Kondoh, Mohamad Alaa Terkawi, Aiko Kume, Mingming Liu, Aaron Edmond Ringo, Huanping Guo, Yang Gao, Seung-Hun Lee, Jixu Li, Paul Franck Adjou Moumouni, Yoshifumi Nishikawa, Hiroshi Suzuki, Ikuo Igarashi, Xuenan Xuan

**Affiliations:** ^1^National Research Center for Protozoan Diseases, Obihiro University of Agriculture and Veterinary Medicine, Obihiro, Japan; ^2^Department of Basic Veterinary Medicine, Obihiro University of Agriculture and Veterinary Medicine, Obihiro, Japan; ^3^Department of Orthopedic Surgery, Hokkaido University, Sapporo, Japan

**Keywords:** *Plasmodium chabaudi*, malaria, *Babesia microti*, babesiosis, infection, macrophages, cross-protection, innate immunity

## Abstract

Malaria and babesiosis, the two primary intraerythrocytic protozoan diseases of humans, have been reported in multiple cases of co-infection in endemic regions. As the geographic range and incidence of arthropod-borne infectious diseases is being affected by climate change, co-infection cases with *Plasmodium* and *Babesia* are likely to increase. The two parasites have been used in experimental settings, where prior infection with *Babesia microti* has been shown to protect against fatal malarial infections in mice and primates. However, the immunological mechanisms behind such phenomena of cross-protection remain unknown. Here, we investigated the effect of a primary *B. microti* infection on the outcome of a lethal *P. chabaudi* challenge infection using a murine model. Simultaneous infection with both pathogens led to high mortality rates in immunocompetent BALB/c mice, similar to control mice infected with *P. chabaudi* alone. On the other hand, mice with various stages of *B. microti* primary infection were thoroughly immune to a subsequent *P. chabaudi* challenge. Protected mice exhibited decreased levels of serum antibodies and pro-inflammatory cytokines during early stages of challenge infection. Mice repeatedly immunized with dead *B. microti* quickly succumbed to *P. chabaudi* infection, despite induction of high antibody responses. Notably, cross-protection was observed in mice lacking functional B and T lymphocytes. When the role of other innate immune effector cells was examined, NK cell-depleted mice with chronic *B. microti* infection were also found to be protected against *P. chabaudi*. Conversely, *in vivo* macrophage depletion rendered the mice vulnerable to *P. chabaudi*. The above results show that the mechanism of cross-protection conferred by *B. microti* against *P. chabaudi* is innate immunity-based, and suggest that it relies predominantly upon the function of macrophages. Further research is needed for elucidating the malaria-suppressing effects of babesiosis, with a vision toward development of novel tools to control malaria.

## Introduction

Malaria persists as one of the biggest global health burdens, despite continuous attempts for its elimination and eradication. *Plasmodium* parasites, the malaria-causing agents, are still endemic in 91 countries and cause over 200 million infection cases every year (World Health Organization., 2016). The host immune response to *Plasmodium* has been a research focal point for decades, as a deeper understanding of the underlying infection mechanism constitutes a major prerequisite for the development of an efficient vaccine. Major strides have been made by malaria researchers toward elucidating the infection and replication machinery of *Plasmodium*. This has been a challenging task, largely due to the complex life cycle of the parasite within the host, which results in the immune system being confronted with multiple morphologically distinct phases of infection- both intracellular and extracellular. It is generally accepted that both humoral and cellular immune responses are required for successful parasite elimination (Langhorne et al., 2002).

Immune memory is possible in malaria, as supported by increased resistance to disease pathogenesis with age (reviewed by Marsh and Kinyanjui, 2006; Doolan et al., 2009). However, complete clinical immunity to malaria requires constant exposure to *Plasmodium* parasites, and even when acquired, it is mostly strain-specific and short-lived (Doolan et al., 2009), although certain levels of cross-protection between different species have been observed in mixed infections due to the existence of common antigens (Collins and Jeffery, 1999; Bruce and Day, 2002). Strikingly, immunity to malaria can also be achieved by means of prior infection with phylogenetically distinct pathogens, a phenomenon immunologists commonly refer to as “heterologous immunity.” Such cases of cross-protection were first demonstrated via experimental murine co-infection models using various *Babesia* and *Plasmodium* species. *Babesia* is a related parasite of the phylum Apicomplexa, widely known for causing babesiosis, a nationally notifiable tick-borne disease in the U.S.A. and emerging zoonosis in parts of Europe and Asia (Homer et al., 2000; Jones et al., 2008; Vannier and Krause, 2012). In particular, *B. microti*, the primary causative agent of human babesiosis, has proven surprisingly effective in providing cross-protection against *Plasmodium* infection in mice (Cox, 1972, 1978; Zivkovic et al., 1984) and primates (van Duivenvoorde et al., 2010).

Beyond laboratory settings, naturally occurring *Babesia* and *Plasmodium* co-infection is possible in areas endemic for both malaria and babesiosis. Unfortunately, human babesiosis tends to be under-diagnosed in malaria-endemic regions, partly because of low parasitemia-mixed infections (Zhou et al., 2013). Additionally, cases of human babesiosis in malaria-endemic regions might be mistaken for *Plasmodium* infections, because of similar clinical presentation and morphology, which make it difficult to distinguish under microscopy. Indeed, babesiosis cases initially misdiagnosed as malaria have been described in Sudan and South Africa (Bush et al., 1990; Abdel et al., 1998). Another important point is that since babesiosis does not respond to antimalarial medication, misidentification as drug-resistant malaria in syndemic areas is likely (Loutan et al., 1993). Despite these obstacles in proper diagnosis, there has been an increasing number of reports of *Babesia* and *Plasmodium* co-infection in humans from syndemic areas, describing rather varied levels of resulting pathogenicity, from light pathology to more severe forms of malaria. The earliest known case of co-infection in humans was reported in 1983, and pertained to a juvenile from the Ivory Coast diagnosed with both *Plasmodium* and *Babesia*, with exacerbated malaria symptoms (Vermeil et al., 1983). More recently, patients with malaria-like symptoms in the Yunnan province- a main malaria endemic area of China- were diagnosed with mixed infections of *B. microti* with *Plasmodium* spp. (Zhou et al., 2013). A patient from the Republic of Korea exhibited prolonged severity of *P. falciparum*-induced hemolytic anemia, possibly due to co-infection with *Babesia sp*. (Na et al., 2014), while an imported babesiosis case involving co-infection with *P. falciparum* was previously described in the country (Ahn, 2010). Finally, in the Democratic Republic of Congo, co-infection with *B. microti* and *P. falciparum* has been diagnosed in minors with a prevalence of 1–6% (Gabrielli et al., 2016). Besides humans, cases of natural co-infection have also been described in closely related primate species; a macaque infected with a *B. microti*-like parasite exhibited suppressed *P. cynomolgi* infection (Wel et al., 2008), while co-infection in lemurs resulted in suppression of malaria infection and pathogenesis (Springer et al., 2015).

Therefore, human and animal cases of *Babesia* and *Plasmodium* co-infection -both in experimental settings and in the wild- suggest that the elicited host immune response has the potential to either ameliorate or exacerbate malaria pathogenicity. Yet, there is still limited insight into the immunology involved during co-infection, and to this day, the mechanisms of interaction between the two parasites within the host remain unknown. In an effort to elucidate this complex phenomenon, we employed a murine infection model for the investigation of the host immune response to blood stage babesiosis and malaria co-infection. Specifically, we examined the effect of various stages of a primary, non-fatal *B. microti* infection on a subsequent lethal *P. chabaudi* challenge infection. Upon observing a robust and long-lasting cross-protection in *B. microti*-infected mice, we further explored the underlying mechanism by testing the efficiency of cross-protection under both the presence and absence of important immune effector cells, namely B and T lymphocytes, Natural Killer (NK) cells and macrophages/monocytes.

## Materials and Methods

### Experimental Animals

Six-weeks-old female BALB/c and C.B-17/Icr-scid/scid (SCID) mice were purchased from CLEA Japan (Tokyo, Japan) and maintained under specific-pathogen-free conditions at the National Research Center for Protozoan Diseases. Food and water were available *ad-libitum*. All animal experiment protocols were approved by the Research Ethics Review Committee of the Obihiro University of Agriculture and Veterinary Medicine (approval number 28–99) and were conducted in accordance with the Standards Relating to the Care and Management of Experimental Animals promulgated by the Obihiro University of Agriculture and Veterinary Medicine of Japan. All experiments were repeated three times to obtain reproducible data.

### Parasite Maintenance and Primary Infection

*Babesia microti* Munich strain and *Plasmodium chabaudi chabaudi* (AS) strain were recovered from laboratory stocks of parasitized RBCs (pRBCs) and used for intraperitoneal (i.p.) passage inoculation in naive mice as previously described (Igarashi et al., 1999). Briefly, primary infection with *B. microti* was performed in five or six mice per group by inoculation of 10^7^ fresh pRBCs in 200–300 μL of PBS-diluted blood obtained from an infected mouse at the height of parasitemia (7–10 d post-infection; 30–40% parasitemia). Mice were infected at the same time with the same parasite inoculum to ensure that all mice received equal numbers of viable parasites. Test mice were infected with *B. microti*, whereas mice of the control group were inoculated with non-parasitized murine erythrocytes (npRBCs) before challenge infection.

### Immunizations With Dead *B. microti*

In order to test whether inoculation with dead *B. microti* can protect against *P. chabaudi* infection, repeated immunizations of mice with glutaraldehyde-fixed *B. microti*-pRBCs and npRBCs were performed as described before (Li et al., 2012). Test mice were vaccinated three times at 14-days intervals with 10^8^ glutaraldehyde-fixed *B. microti*-pRBCs, while control mice were injected with glutaraldehyde-fixed npRBCs. Two weeks after the last inoculation, blood samples were drawn from all mice and used in an enzyme-linked immunosorbent assay (ELISA) to determine the specific humoral response to *B. microti*. Control and test mice were then challenge-infected with *P. chabaudi*.

### Detection of Specific Antibodies to *B. microti* P32 and Assessment of Cross-Reactivity With Crude *P. chabaudi* Antigen

The antibody profiles of mice immunized with either live or dead *B. microti* were examined 14 days after the final immunization. Antibody levels against *B. microti* were determined by using recombinant *B. microti* P32 (rBmP32) specific antigen in an ELISA assay, as previously described (Li et al., 2012). In brief, ELISA plates were coated with rBmP32 and incubated with mouse sera, then with HRP-conjugated goat anti-mouse IgGs and IgM as a secondary antibody. Additionally, the possible cross-reactivity of antibodies of *B. microti*-immunized mice against *P. chabaudi* was examined. The same samples were used in an ELISA assay where a lysate of the erythrocytic stages of *P. chabaudi* was used as coating antigen, as previously described (Seixas and Ostler, 2005). Briefly, RBCs from *P. chabaudi*-infected mice were chemically lysed, and the intact *P. chabaudi* parasites were ultra-sonicated. The protein content of the *P. chabaudi* lysate was measured using NanoDrop 2000 (Thermo Fisher Scientific, USA) and ELISA plates were coated with the crude malaria antigen, incubated with the serum samples, and then with HRP-conjugated goat anti-mouse IgG and IgM as a secondary antibody. Hyper-immune serum obtained from mice challenged with a high dose of *P. chabaudi*-pRBCs was used as positive control, and serum from mice inoculated with npRBCs was used as a negative control. The optical density was measured with an MTP-500 microplate reader (Corona Electric, Japan) at 415 nm.

### *P. chabaudi* Challenge Infection

To examine the effect of a *B. microti* infection against a subsequent *P. chabaudi* challenge, test and control mice were challenge infected with 10^5^ *P. chabaudi*-pRBCs. Challenge infection of the immunocompetent BALB/c mice that were immunized with live *B. microti* was performed at days 0, 7, 14, 28, or 56 post primary infection. For the mice injected with dead *B. microti*, challenge infection was executed 14 days after the last inoculation. Lastly, SCID mice were challenged with *P. chabaudi* at day 28 post primary infection.

### Determination of Parasitemia, Hematocrit Values, Body Weight, and Survival Rates

Parasitemia levels, hematocrit values, body weight, and survival rates of experimental mice were regularly monitored. For estimation of parasitemia, at least 10^3^ erythrocytes were examined under an oil immersion microscope from Giemsa-stained thin blood smears prepared from blood collected daily by tail snip. For the evaluation of hematological kinetics, 10 μl of blood collected from each mouse every 2 days was used for a full blood cell count carried out on an automatic cell counter (Nihon Kohden, Japan). All mice were checked on a daily basis for any mortalities and body weight changes for 28 days after challenge infection.

### Detection of Malaria-Specific Antibodies Using Crude *P. chabaudi* Antigen

Test mice either acutely or chronically infected with *B. microti* as well as control mice were challenge-infected with *P. chabaudi*, and serum samples were obtained at days 2, 4, and 6 post challenge infection. Following an ELISA protocol similar to out cross-reactivity assay described above, *P. chabaudi*-specific IgG, IgG1, and IgM were measured against a coating antigen made of a *P. chabaudi* erythrocytic stage lysate.

### Detection of Serum Cytokines

At days 2, 4, and 6 post challenge infection with *P. chabaudi*, serum was obtained from test and control mice. Commercial ELISA kits were used to determine the cytokine concentrations by means of respective standard curves prepared with known concentrations of mouse recombinant IFN-γ, IL-2, IL-10, IL-12+p40, and TNF-α (Thermo Fischer Scientific, USA), according to the manufacturer's instructions.

### *In vivo* Depletion of NK Cells and Macrophages/Monocytes

To study the role of NK cells in the *B. microti*-conferred cross-protection against *P. chabaudi*, NK cells were depleted *in vivo* in BALB/c mice chronically infected with *B. microti*. In brief, 50 μl of anti-asialo GM-1 antibody (Thermo Fischer Scientific, USA) in 200 μl of PBS were given to test mice by intra-peritoneal injection on days −2, +3, and +6 with regard to *P. chabaudi* infection, following a protocol previously described by (Couper et al., 2007). Control mice were administered equal volume of control rabbit antibody diluted in 200 μl of PBS. The effective depletion of NK cells in the spleen was confirmed by flow cytometry using anti-mouse DX5 antibody (Thermo Fischer Scientific, USA).

In separate experiments, the role of macrophages in cross-protection was examined by *in vivo* systemic depletion of macrophages in BALB/c mice as previously described (Couper et al., 2007). Macrophage depletion was carried out in test mice with chronic *B. microti* infection via i.p. administration of 300 μl of clodronate liposomes 2 days before and 3 days after challenge infection with *P. chabaudi*, which was performed at day 28 post primary infection. Mock mice were administered 300 μl of PBS encapsulated in liposomes, while control mice were treated with equal volume of sterile PBS. Clodronate and PBS liposomes were purchased from www.clodronateliposomes.org (Haarlem, the Netherlands).

### Flow Cytometry Assays

Successful *in vivo* depletion of NK cells and macrophages/monocytes in mice was confirmed via flow cytometry assays. Briefly, for macrophage depletion confirmation, cells derived from the peritoneal fluid of three mice per group were isolated 5 days after the final treatment with CLL or PL and resuspended in 0.5% bovine serum albumin diluted in cold phosphate-buffered saline (PBS). Next, the cells were incubated with phycoerythrin (PE)-labeled anti-mouse F4/80 (BM8) mAb (Thermo Fischer Scientific, USA) on ice for a half hour. The cells were then washed thrice with cold PBS and analyzed on an Epics XL flow cytometer (Beckman Coulter, Pasadena, CA, USA). For confirmation of NK cell depletion, splenocytes of three mice per group were obtained 2 days after the last injection with anti-asialo GM-1 antibody or control rabbit antibody and incubated with fluorescein isothiocyanate (FITC)-labeled anti-mouse CD49b/Pan-NK cell (DX5) mAb (Thermo Fischer Scientific, USA). For all experiments, ten thousand cells were detected in each sample. Non-specific staining was avoided with the use isotype controls established using matched fluorescence-labeled isotype control antibodies.

### Histopathology of Spleen Sections

The efficacy of macrophage depletion in mice was additionally verified by histopathological examination of spleen sections as described previously (Tanaka et al., 2013). Briefly, spleens were harvested from test mice injected with clodronate and control mice inoculated with PBS liposomes or PBS, and the tissues were fixed in 4% (v/v) paraformaldehyde, embedded in paraffin, and then stained with hematoxylin and eosin.

### Immunofluorescence Antibody Test (IFAT)

Since both *B. microti* and *P. chabaudi* replicate within erythrocytes and share many morphological features, differentiation of the two parasites via conventional staining/standard light microscopy can be challenging. In order to safely differentiate between the two parasitic infections, standard IFAT was performed using anti-sera obtained from mice repeatedly challenged with *B. microti* and *P. chabaudi*. Briefly, IFAT slides were coated with pRBCs obtained at day 6 post challenge infection with *P. chabaudi* from both immunocompetent and SCID mice chronically (28 days) infected with *B. microti*. The slides were fixed and incubated with either *B. microti* or *P. chabaudi* anti-serum, and then with Alexa-Fluor® 680- or Alexa-Fluor® 488-conjugated goat anti-mouse IgG (Molecular Probes, USA) secondary antibody, respectively. The slides were examined using a fluorescent microscope (E400 Eclipse, Nikon, Japan).

### Statistical Analysis

GraphPad Prism 6 software (GraphPad Software, USA) was used for all statistical analyses. Following computation of the means of all variables, one-way analysis of variance and Tukey's multiple-comparison test were used for pairwise comparison of data from the multiple groups. A Kaplan-Meier non-parametric model was employed for survival analyses for significant differences. Results were considered statistically significant when the *P* < 0.05.

## Results

### Mice Primarily Infected With *B. microti* Are Completely Protected Against Lethal *P. chabaudi*

**Figure 1:**
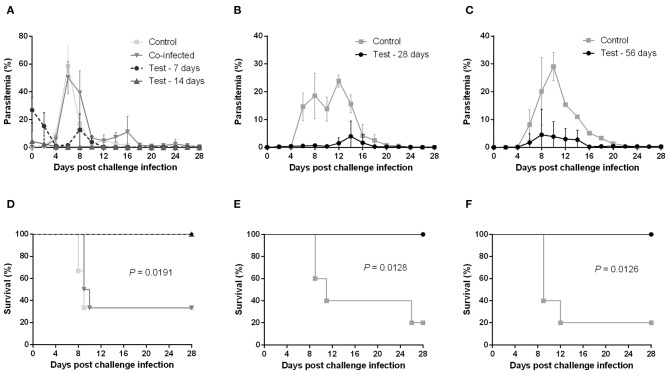
Course of *P. chabaudi* challenge infection in BALB/c mice undergoing different stages of primary *B. microti* infection. Test BALB/c mice were initially infected with *B. microti* and then challenge-infected with *P. chabaudi* at different time points (on days 0, 7, 14, 28, or 56) post primary infection. Control mice received *P. chabaudi* alone. Courses of parasitemia **(A–C)** and survival rates **(D–F)** of control and test mice are presented. Results are expressed as mean percent parasitemia values ± the standard deviation (SD) of five or six mice. Mean percent parasitemia values are calculated from individual percent parasitemia levels taken from all surviving mice at each specific time point. Experiment shown is representative of three independent experiments. Experiments of challenge infection on days 0, 7, and 14 **(A,D)** belonged to a single cohort and as such share the same control group.

Mice primarily infected with *B. microti* exhibited temporary high parasitemia, which reached its peak around day 10 and gradually decreased to very low levels within 4 weeks. Yet, *B. microti*-pRBCs could still be observed in blood smears for at least 56 days after primary infection. We were thus able to confirm a state of chronic, low-level *B. microti*-induced parasitemia in mice exhibiting no other obvious clinical symptoms. This state of persistent infection also explains small peaks of recurring parasitemia we observed in some mice chronically infected with *B. microti*. To determine whether co-infection and various stages of *B. microti* infection can protect against a lethal *P. chabaudi* challenge, mice were initially injected with *B. microti*, then challenged with *P. chabaudi* at different time points: simultaneously with *B. microti* (day 0), at the acute stage (day 7), at the resolving stage (day 14), and at the chronic stage of primary *B. microti* infection (days 28 and 56). Interestingly, mice simultaneously infected with both parasites exhibited a rapid increase in parasitemia and two thirds of them eventually succumbed to the infection, similar to control mice infected with *P. chabaudi* alone (Figures 1A,D). Moreover, simultaneously infected mice underwent significant body weight loss and hematocrit decrease, starting at 6 days post infection (Supplementary Figures 1A,D). Conversely, mice challenged at the acute, resolving, and chronic stages of *B. microti* infection were fully resistant to *P. chabaudi*, as evidenced by 100% survival rates and significantly lower levels of parasitemia (Figures 1A–F). In the 28th-day-challenge experiment, control mice exhibited a slight decline in parasitemia before eventually reaching a delayed peak at day 12 (Figure 1B). Potential reasons for this different pattern include a handling error in the equal distribution of *P. chabaudi* inoculum, or use of parasites slightly attenuated from extended cold storage. Nevertheless, a distinct response to the challenge infection was still evident between test and control mice. Furthermore, mice primarily infected with *B. microti* did not exhibit any substantial hematocrit fluctuations, nor did they experience body weight loss after the challenge infection, as opposed to control mice (Supplementary Figures 1A–F). IFAT for pRBCs collected 6 days after the *P. chabaudi* challenge infection from mice chronically infected with *B. microti* revealed that most pRBCs were infected with *P. chabaudi*, although low numbers of *B. microti*-pRBCs were still observable (Supplementary Figure 2). These results demonstrate that primary infection -but not co-infection- with *B. microti* grants mice complete cross-protection against a lethal *P. chabaudi* infection.

### Immunization With Dead *B. microti* Elicits a Humoral Response but Does Not Confer Protection Against *P. chabaudi*

**Figure 2:**
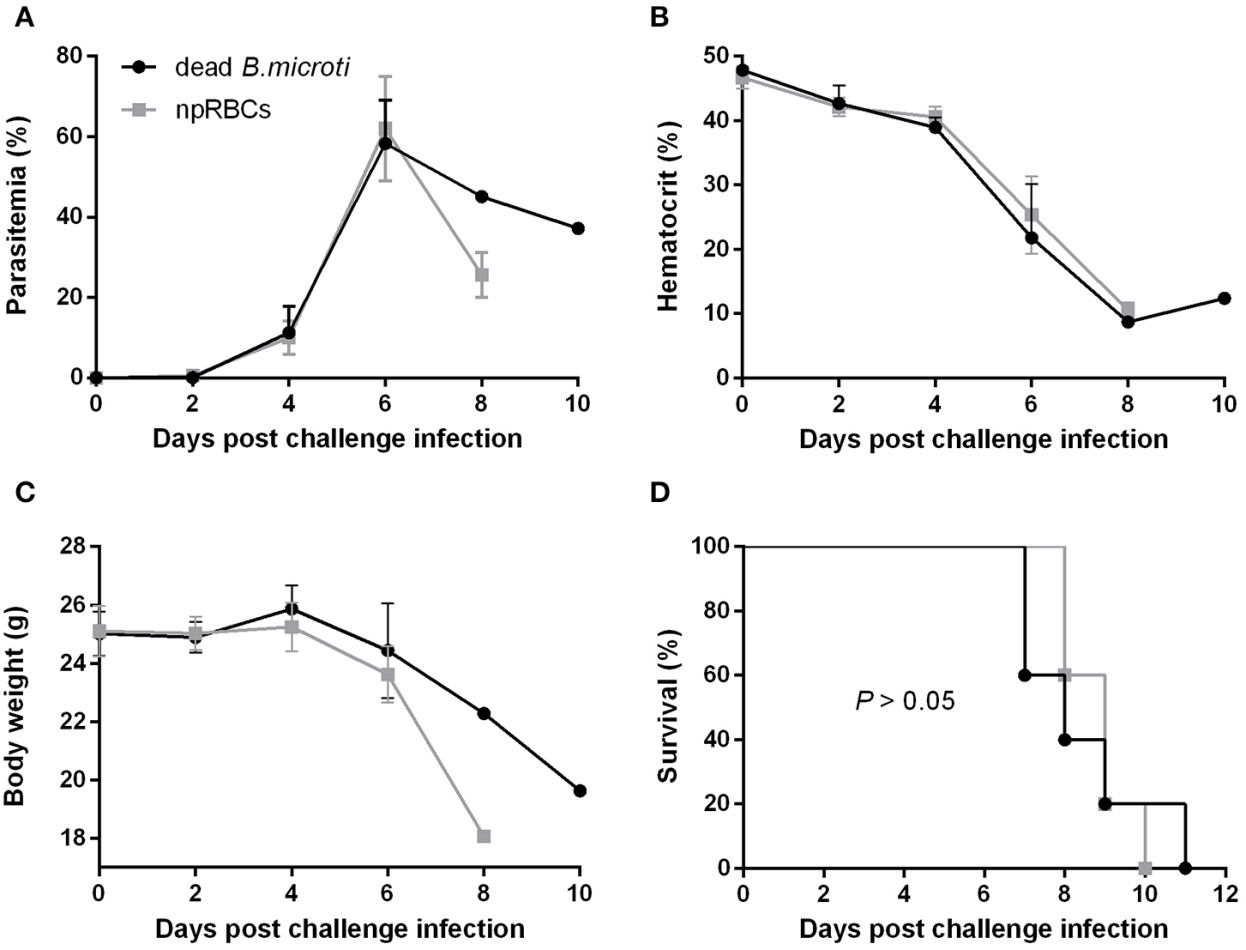
Course of *P. chabaudi* challenge infection in BALB/c mice immunized with dead *B. microti*. BALB/c mice were immunized three times at 2-weeks intervals with either glutaraldehyde-fixed *B. microti*-pRBCs or glutaraldehyde-fixed non-parasitized RBCs (npRBCs). Two weeks after the last immunization, all mice were challenge-infected with *P. chabaudi*. Parasitemia **(A)**, hematocrit **(B)**, body weight **(C)**, and survival rates **(D)** were monitored over a period of 28 days after challenge infection. Results are expressed as mean values ± the SD of five mice. Mean percent parasitemia, hematocrit, and body weight values are calculated from individual values taken from all surviving mice at each specific time point. Data presented are from one experiment representative of three.

To examine whether immunization with dead *B. microti* parasites can confer cross-protection similar to the live parasites, mice were subjected to immunizations using glutaraldehyde-fixed *B. microti*-pRBCs. Both mice immunized with dead *B. microti* and control, non-immunized mice showed prompt increases in parasitemia after challenge infection with *P. chabaudi*, starting at day 2 and reaching a peak of over 60% by day 6 (Figure 2A). Furthermore, both groups experienced a sharp reduction in the hematocrit, and the rise in parasitemia was accompanied by substantial body weight loss (Figures 2B,C). Eventually, all mice succumbed to *P. chabaudi* infection and died within 11 days from the challenge (Figure 2D). Interestingly, mice immunized with dead *B. microti* produced high titers of specific IgG1 against rBmP32, but did not produce significant amounts of IgG2a or IgM (Supplementary Figure 3A). Next, we investigated the ability of specific antibodies raised against both live and dead *B. microti* parasites to cross-react with crude *P. chabaudi* antigens. Notably, sera obtained 14 days after immunization from mice injected with either live or dead *B. microti* failed to exhibit any significant levels of cross-reactivity with *P. chabaudi*, exhibiting the same levels of IgG and IgM as the npRBCs-inoculated controls (Supplementary Figure 3B). To confirm the validity of these results, a positive control serum sample from *P. chabaudi*-infected mice was tested against the same *P. chabaudi* lysate, and high titers of both IgG and IgM were successfully detected (Supplementary Figure 3B). These results strongly indicate that even though immunization with dead *B. microti* is able to elicit a humoral response in mice, it fails to protect them against a *P. chabaudi* challenge infection. Furthermore, *B. microti*- specific antibodies from protected mice are unable to recognize and react with *P. chabaudi* antigens.

### Protected Mice Have Low Levels of Antibodies Against *P. chabaudi* and Cytokines Post Challenge Infection

**Figure 3:**
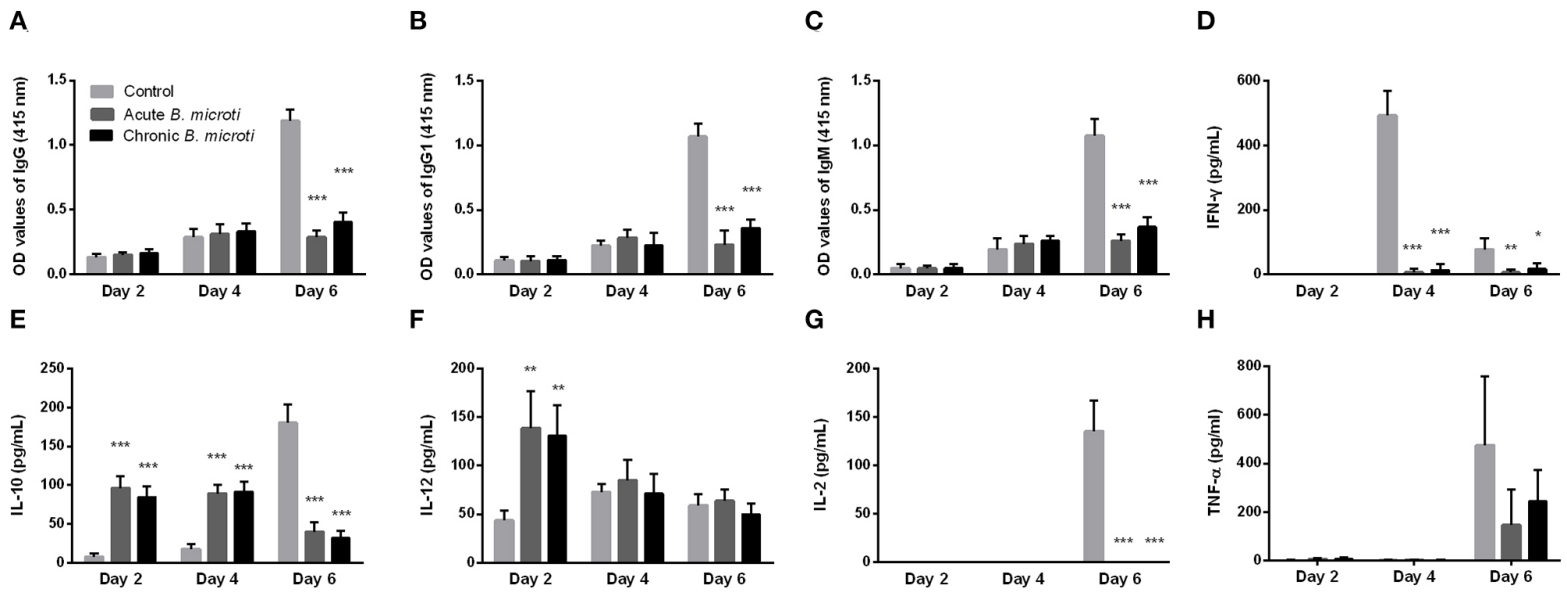
The kinetics of serum anti-*P. chabaudi* antibodies and cytokines of protected and susceptible mice after *P. chabaudi* challenge infection. Test mice were initially infected with *B. microti* and then challenged with *P. chabaudi* on days 7 (acute group) or 28 (chronic group) post primary infection. Control mice received *P. chabaudi* alone. On days 2, 4, and 6 after challenge infection the levels of IgG **(A)**, IgG1 **(B)**, IgM **(C)**, IFN-γ **(D)**, IL-10 **(E)**, IL-12 **(F)**, IL-2 **(G)**, and TNF-α **(H)** were determined. For detection of serum antibodies against *P. chabaudi*, crude *P. chabaudi* lysate was used as coating antigen in ELISA assays. Asterisks indicate statistically significant differences (*P < 0.05; **P < 0.005, and ***P < 0.0001 compared to control mice). Results are expressed as mean values ± the SD of five mice.

Subsequently, the levels of serum antibody and cytokines of mice with acute and chronic *B. microti* infection were measured at days 2, 4, and 6 post challenge infection with *P. chabaudi*. The results were then compared with control mice, which received no prior infection with *B. microti*. Malaria-specific antibodies were detected in substantial levels at day 6, however IgG, IgG1, and IgM levels were significantly lower in *B. microti*-infected mice (Figures 3A–C). Regarding cytokine production, IFN-γ levels were undetectable on day 2, but significantly lower in acutely and chronically *B. microti*-infected mice than in control mice (Figure 3D). In contrast, IL-10 was detected in higher levels in *B. microti*-infected mice on days 2 and 4, however control mice had higher serum IL-10 levels 6 days after the challenge infection (Figure 3E). *B. microti*-infected mice had increased IL-12 levels compared to control mice at day 2, but these levels were not significantly different at days 4 and 6 (Figure 3F). Moreover, IL-2 was significantly lower in test mice at day 6, when the cytokine was first detected (Figure 3G). Lastly, levels of TNF-α remains below detection levels until day 6 post challenge infection, at which point no significant difference was detected between the test and control groups (Figure 3H). The above results show that both acutely and chronically *B. microti*-infected mice, previously shown to be protected against *P. chabaudi* challenge, regulate their antibody and cytokine production in a substantially different way in response to a lethal *P. chabaudi* infection when compared to susceptible control mice.

### Absence of B and T Lymphocytes Does Not Impede the *B. microti*-Conferred Protection Against *P. chabaudi*

**Figure 4:**
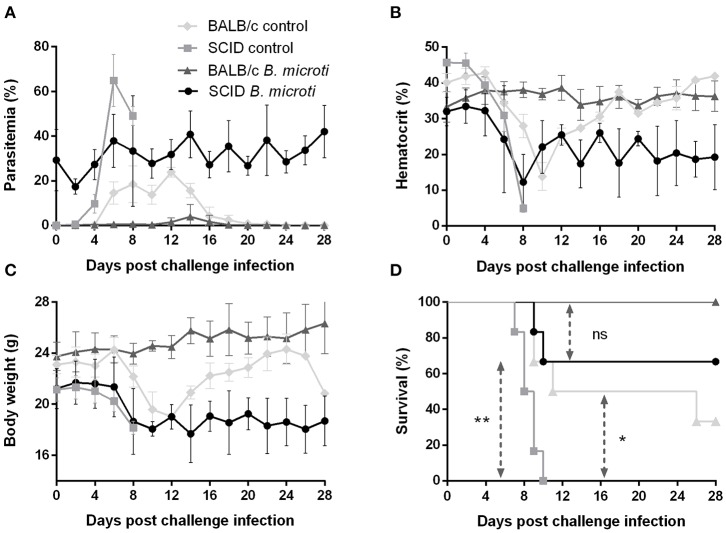
Course of *P. chabaudi* challenge infection in SCID and BALB/c mice chronically infected with *B. microti*. Test SCID and BALB/c mice were initially infected with *B. microti* and then challenged with *P. chabaudi* on day 28 after primary infection. Control SCID and BALB/c mice received *P. chabaudi* alone. Parasitemia **(A)**, hematocrit **(B)**, body weight **(C)**, and survival rates **(D)** were monitored over a period of 28 days after challenge infection with *P. chabaudi*. Results are expressed as mean values ± the SD of six mice. Mean percent parasitemia, hematocrit, and body weight values are calculated from individual values taken from all surviving mice at each specific time point. Asterisks indicate statistically significant differences (*P < 0.05 and **P < 0.005 between different groups. *Ns* = *P* > 0.05).

The role of the adaptive immune response in the cross-protection was investigated by carrying out a *P. chabaudi* challenge infection in SCID mice on the BALB/c background, which are severely deficient in functional B and T lymphocytes. The course of challenge infection was compared between test SCID mice chronically infected with *B. microti* and control SCID mice not immunized with *B. microti*. Test SCID mice demonstrated *B. microti*-induced parasitemia which persisted in high levels (20–30%) for at least 28 days, at which point they were challenged with *P. chabaudi* (Figure 4A). Most SCID mice with chronic *B. microti* infection survived the challenge infection, although they consistently maintained high parasitemia characterized by fluctuation (Figure 4A). Examination by IFAT revealed that pRBCs of test SCID mice after challenge infection were predominantly infected with *P. chabaudi*, although *B. microti*-pRBCs were also visible (Supplementary Figures 2J–L). Remarkably, *B. microti*-infected SCID mice went through similar hematocrit and body weight declines as did control SCID mice, without being able to recover fully over the course of 28 days (Figures 4B,C). In stark contrast, control SCID mice exhibited a sharper increase in parasitemia and succumbed to *P. chabaudi* infection within 10 days (Figures 4A,D). Notably, the survival rates of control SCID mice after *P. chabaudi* infection were significantly lower (*P* = 0.0176) than those of immunocompetent control mice, demonstrating the role of lymphocytes and the adaptive immune system in combating *P. chabaudi* infection (Figure 4D). Protection conferred by chronic infection with *B. microti* was lower in SCID mice (66% survival rate) in comparison to BALB/c mice (100% survival rate), but reduction was statistically insignificant (*P* = 0.1396) (Figure 4D). Importantly, the majority of SCID mice chronically infected with *B. microti* survived the challenge with *P. chabaudi*, whereas all non-immunized SCID mice quickly succumbed to the malarial infection (*P* = 0.0084). These results demonstrate that protection elicited by primary *B. microti* infection does not rely upon functional B and T lymphocytes, strongly suggesting a crucial role for the innate immune system in the mechanism underlying cross-protection.

### Lack of Macrophages/Monocytes but Not NK Cells Impairs the *B. microti*-Conferred Protection in BALB/c Mice

**Figure 5:**
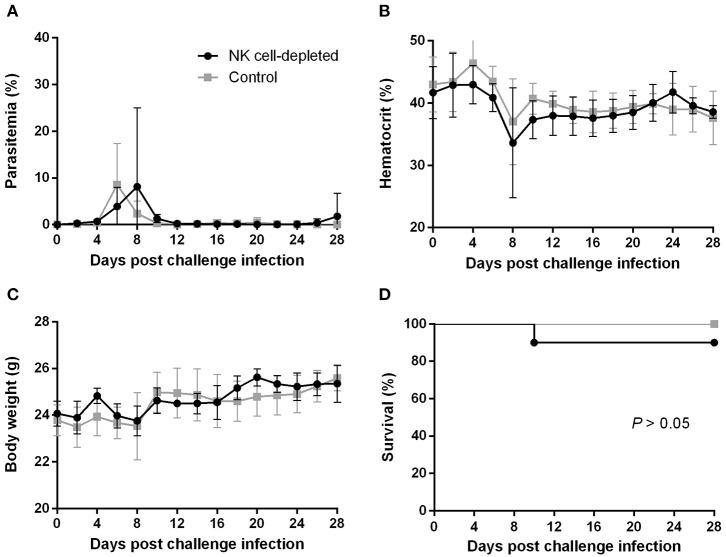
Effect of NK cell depletion on the course of *P. chabaudi* challenge infection in BALB/c mice chronically infected with *B. microti*. BALB/c mice with chronic *B. microti* infection were treated with anti-asialo-GM1 antibody for NK cell depletion (test group) or control rabbit antibody (control group). Parasitemia **(A)**, hematocrit **(B)**, body weight **(C)**, and survival rates **(D)** were monitored over a period of 28 days after challenge infection with *P. chabaudi*. Results are expressed as mean values ± the SD of six mice. Mean percent parasitemia, hematocrit, and body weight values are calculated from individual values taken from all surviving mice at each specific time point.

**Figure 6:**
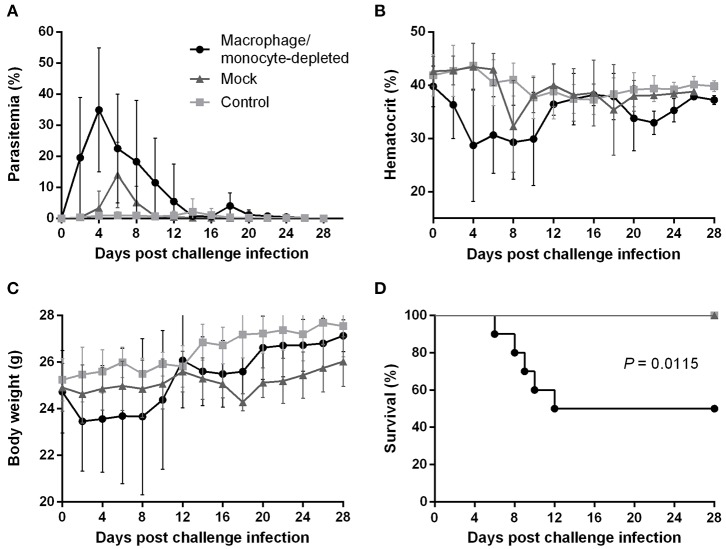
Effect of macrophage/monocyte depletion on the course of *P. chabaudi* challenge infection in BALB/c mice chronically infected with *B. microti*. BALB/c mice with chronic *B. microti* infection were treated with clodronate liposome for macrophage/monocyte depletion (test group), while mock mice received PBS-liposome and control mice received sterile PBS. Parasitemia **(A)**, hematocrit **(B)**, body weight **(C)**, and survival rates **(D)** were monitored over a period of 28 days after challenge infection with *P. chabaudi*. Results are expressed as mean values ± the SD of 10 mice. Mean percent parasitemia, hematocrit, and body weight values are calculated from individual values taken from all surviving mice at each specific time point.

To better understand how the innate immune system contributes to the *B. microti*-conferred cross-protection against *P. chabaudi*, the next step was to examine whether cross-protection would be diminished in the absence of important immune effector cells, namely NK cells and macrophages/monocytes. To do this, in two separate sets of experiments BALB/c mice chronically infected with *B. microti* were depleted of either NK cells or macrophages/monocytes by means of i.p. administration of anti-asialo GM1 antibody and clodronate liposomes, respectively. *In vivo* depletion of NK cells in mice chronically infected with *B. microti* had no noticeable effect on the progression of challenge infection, given that no substantial changes were observed in parasitemia levels, body weight or hematocrit values in test mice compared to their control counterparts (Figures 5A–C). Accordingly, there was no notable difference (*P* = 0.3173) in survival rates between control and NK cell-depleted mice (Figure 5D). In sharp contrast, in our macrophage depletion experiments, clodronate liposome-treated mice developed significantly higher levels of parasitemia when compared to mock mice that received PBS liposomes and control mice injected with PBS (Figure 6A). Accordingly, macrophage-depleted mice showed a temporary yet considerable decrease in hematocrit and body weight within the first 2 weeks, whereas control mice maintained these parameters throughout the course of challenge infection (Figures 6B,C). Ultimately, 50% of the macrophage-depleted mice perished within the 2nd week of challenge infection with *P. chabaudi*, a significant decrease (*P* = 0.0115) compared to the non-depleted, control mice (Figure 6D). The successful depletion of macrophages was confirmed by flow cytometry as well as histopathological assays of the spleen. Examination of spleen sections of control mice chronically infected with *B. microti* revealed expanded red and white pulp compartments, yet distinct marginal zones. On the other hand, macrophage-depleted mice displayed strong architectural disorganization, signified by a loss of cells in the marginal zone (Supplementary Figure 4). Combined, these results strongly indicate a crucial role for macrophages, but not NK cells, in the cross-protection induced by *B. microti* primary infection against *P. chabaudi*.

## Discussion

Studies of host-pathogen interaction in animals typically focus on single infections in immunologically naïve hosts (Page et al., 2006). Despite this being a sensible approach to mechanistically elucidate complex immune pathways, co-infections with several pathogens are common in nature. This results in intricate antagonistic or synergistic interactions within the host, which can in turn affect each parasite's abundance and decisively factor in the risk of infection (Pedersen and Fenton, 2007; Telfer et al., 2008). Therefore, it is vital to shed light into the occurrence of parasitic co-infections and investigate the underlying mechanisms of interaction within the host, especially when it comes to devastating human pathogens. *Plasmodium chabaudi* has been a valuable tool in the investigation of malaria immunopathology (Langhorne et al., 2008, reviewed by Stephens et al., 2012), and was thus chosen for our co-infection model. Moreover, considering the increasing number of reports of human co-infection with *Plasmodium* and *Babesia* in recent years (Ahn, 2010; Zhou et al., 2013; Na et al., 2014; Gabrielli et al., 2016), the second pathogen employed was *Babesia microti*, an emerging zoonotic parasite and the leading cause of babesiosis in humans.

Historically, the phenomenon of heterologous immunity, first observed between the smallpox and cowpox viruses during the groundbreaking work of Edward Jenner, has been primarily studied using viral infection models. As a rule, cross-protection is achieved either specifically by cross-reacting T cells and antibodies (Selin et al., 2006), or in a non-specific manner by macrophages activated during primary infection that create a heightened state of innate immunity to subsequent, even non-viral, infections (Barton et al., 2007; Welsh et al., 2010). Generally, heterologous immunity is not as effective or long-lasting as homologous immunity, yet, it can still result in a less severe infection (Selin et al., 1998). The mechanism behind heterologous immunity observed between various protozoan parasites remains largely unknown.

In this study, we set out to examine the effect of primary *B. microti* infection on a subsequent *P. chabaudi* challenge in mice. We first showed that immunocompetent mice in various stages of *B. microti* infection were completely resistant to lethal infection with *P. chabaudi*. Chronic *B. microti* infection lasting for at least 56 days was confirmed by microscopy, and is in agreement with past studies showing chronic, low-parasitemic *B. microti* infection persisting for 6 months in BALB/c mice (Welc-Faleciak et al., 2007), as well as in wild rodents (Pawelczyk et al., 2004). In fact, persistent and sometimes recrudescent babesiosis has also been reported in humans, with an asymptomatic carrier state lasting up to 2 years in immunocompetent individuals, while more prolonged and severe infection is common in asplenic patients (reviewed by Bloch et al., 2019). The spleen, main site of immune response to babesiosis, undergoes disruption of its normal architecture; splenomegaly, coupled with enlargement of the white and red pulp regions, is typical of murine babesiosis (Coleman et al., 2005; Djokic et al., 2018a,b) as well as other intraerythrocytic infections, including malaria (Leisewitz et al., 2004; Buffet et al., 2011). Such histopathological changes are at least partially attributed to the expansion of macrophages, which help destroy *Babesia*-pRBCs and ultimately determine the outcome of *B. microti* infection (Terkawi et al., 2015; Djokic et al., 2018a). Erosion of the marginal zone is prominent during resolving infection (Djokic et al., 2018a), but was not detectable at the chronic stage in our experiments. Failure in parasite clearance even when host immune function is intact could be the result of multiple effective immune evasion strategies employed by *Babesia* spp. One such mechanism, involves adherence to the vascular endothelium via molecules expressed on the surface of pRBCs (Allred and Al-Khedery, 2004), allowing the parasite's replication and invasion of other erythrocytes without having to circulate through the spleen. In evolutionary terms, persistence of infection can help increase the parasite's fitness by maximizing the probability of transmission between the reservoir host and the vector (Allred, 2003).

Our findings parallel previous studies, in which chronic *B. microti* infection in monkeys conferred resistance against *P. cynomolgi* (van Duivenvoorde et al., 2010), and mice infected with non-lethal malaria survived lethal *P. berghei* subsequent and simultaneous infections (Niikura et al., 2008). In contrast, our results show that mice simultaneously infected with both *B. microti* and *P. chabaudi* were not protected, mirroring a previous study, where primary *B. microti* infection protected mice against a lethal *B. rodhaini* challenge, but not when infection was simultaneous (Li et al., 2012). Interestingly, simultaneous inoculation with *B. microti* has even been linked to more severe disease manifestations compared to single infection, as shown in murine Lyme disease co-infection models (Moro et al., 2002; Djokic et al., 2019). Failure in protection in our experiments could be due to *P. chabaudi* initiating a rapid wave of parasitemia in the blood stream, before *B. microti* can initiate appropriate immune responses that subdue malaria pathogenesis. Furthermore, our protocol of repeated immunizations with dead *B. microti* did not confer protection against *P. chabaudi*, as opposed to pre-infection with live *B. microti*. In past studies, inoculation with live *Plasmodium*-pRBCs has prompted a more efficient response compared to dead *Plasmodium* parasites (Waterfall et al., 1998; Artavanis-Tsakonas and Riley, 2002), which might hold true for *Babesia*, as well. Immunization with either live or dead *B. microti* led to generation of high levels of specific antibody against the parasite. However, in neither case did the specific antibodies against *B. microti* cross-react *in vitro* with crude *P. chabaudi* antigens. This suggests that the two parasites share no common antigenic epitopes, indicating that any cross-protective mechanism is antibody-independent. In further support of this, protected *B. microti*-infected mice produced pointedly lower levels of anti-*P. chabaudi* antibodies. This could be due to *B. microti*-induced splenic complications which reduce lymphocyte function and antibody production, as previously shown during murine *B. microti*- *Borrelia burgdorferi* co-infection (Bhanot and Parveen, 2019; Djokic et al., 2019).

As is the case with various intraerythrocytic pathogens, protective immunity to *Babesia* and *Plasmodium* requires an efficient Th1 response during early infection in order to control blood-stage replication (Langhorne et al., 1989; Li et al., 2012). *P. chabaudi* is non-fatal to Th1-inclined mouse strains (Stevenson et al., 1982; Wunderlich et al., 1988), but lethal to the Th2-biased BALB/c strain (Scott et al., 1989; Watanabe et al., 2004). Mice susceptible to malaria are generally incapable of mounting a targeted and effective immune response. Paradoxically, such susceptibility may be caused by the triggering of overly potent innate responses, or pro-inflammatory “cytokine storms,” resulting in systemic inflammation and death (Cross and Langhorne, 1998; Riley, 1999). It is therefore accepted that appropriate balance of pro-inflammatory cytokines to enable parasite clearance but avoid pathology is essential during early *Plasmodium* infection. Similarly, resistance to murine babesiosis is associated with early induction of IL-12 and IFN-γ, assisting with parasitemia control at the acute stage (Igarashi et al., 1999; Aguilar-Delfin et al., 2003). Nevertheless, a systemic pro-inflammatory cytokine response is not needed for parasite clearance (Skariah et al., 2017). Instead, *B. microti* causes a sustained increase in the levels of serum IL-10, which plays a major role in the resolution of infection (Jeong et al., 2012; Djokic et al., 2018a). As a key regulatory cytokine, IL-10 may suppress over-expression of IFN-γ and TNF-α while permitting an immune response robust enough to eradicate infection (Trinchieri, 2007; Couper et al., 2008; Redpath et al., 2014). The above are in agreement with our findings, where protected mice displayed decreased levels of IFN-γ, no strong TNF-α production, and higher levels of IL-10 after challenge infection with *P. chabaudi*. Overproduction of IFN-γ and IL-12 has been linked to pathology in malaria models (Yoshimoto et al., 1998; Engwerda et al., 2002). Inversely, production of IL-10 during early *Plasmodium* infection inhibits Th1 immune responses (Wu et al., 2007), while its absence causes more severe disease due to overproduction of TNF-α (Li et al., 1999, 2003). Taken together, our results seem to indicate that the protection conferred by *B. microti* against *P. chabaudi* lies in guiding the BALB/c immune system toward an appropriate immune response by helping overcome genetic Th2 bias while also avoiding an exaggerated systemic inflammation.

Protective immunity conferred by *B. microti* against *P. chabaudi* was largely independent of B and T lymphocytes. Antibodies have been long known to be crucial for the final elimination of *P. chabaudi* (Meding and Langhorne, 1991), however some control of parasitemia during acute infection is possible in their absence (von der Weid et al., 1996). Indeed, we showed here that control of the acute stage of *P. chabaudi* challenge precedes the production of noticeable levels of specific antibody. T cells are considered key in modulating human immunity to malaria due to their ability to produce immunoregulatory cytokines (Todryk et al., 2008). Specifically, CD4+ T cells can activate macrophages to produce TNF-α and cytotoxic soluble mediators through the release of inflammatory cytokines (Stevenson et al., 1995; Good and Doolan, 1999), and can rescue immunodeficient mice from fatal *P. chabaudi* infection (Stephens et al., 2005). Importantly, T cells, particularly CD4+ T cells, are critical for clearance of *B. microti* infection (Igarashi et al., 1999; Clawson et al., 2002; Skariah et al., 2017), which explains why SCID mice in our experiments experienced prolonged parasitemia after *B. microti* primary infection. This was followed by fluctuating levels of mixed parasitemia after challenge with *P. chabaudi*, confirming the inability of SCID mice to clear co-infection in the absence of adaptive immunity. Yet, as evidenced by survival of most SCID mice, innate immunity, primed by *B. microti*, was sufficient in combating lethal malaria pathogenicity. As with babesiosis, the spleen is fundamental in developing immune responses to blood-stage malaria, with highly phagocytic red pulp macrophages on the first lines of defense (Yadava et al., 1996; Engwerda et al., 2005). *B. microti*-induced splenomegaly, accompanied by boosted macrophage phagocytosis, may have a protective effect against *P. chabaudi*. There is indication that spleen enlargement affects its filtering capacity: acute malaria patients with splenomegaly accelerated clearance of erythrocytes as opposed to patients with normal spleen sizes (Wyler et al., 1981; Looareesuwan et al., 1987). We thus propose that *P. chabaudi* challenge infection in SCID mice chronically infected with *B. microti* is met with an enhanced spleen capacity for filtering pRBCs, which however cannot lead to elimination of co-infection in the absence of B and T cells. The resulting loop of parasite replication in the circulation and accelerated removal in the spleen is evident in the persistent and fluctuating parasitemia. Our finding that *B. microti*-infected SCID mice are largely protected against *P. chabaudi* unequivocally signifies that the mechanism of cross-protection is antibody- and T-cell independent.

The above observation clearly points to a central role for the innate immune system in the cross-protective mechanism. Innate immunity is crucial for babesiosis resistance in mice (Aguilar-Delfin et al., 2001), however the question as to how it may contribute to cross-protection remains. Past studies indicate that NK cells are vital for the establishment of protective immunity to malaria (Mohan et al., 1997; Su and Stevenson, 2000). However, we showed here that lack of NK cells does not impair cross-protection. On the other hand, *in vivo* systemic depletion of macrophages/ monocytes greatly reduced survival rates of *B. microti*-infected mice following *P. chabaudi* challenge infection. As previously described (Terkawi et al., 2015), macrophage depletion resulted in observable cell loss in the red pulp and shrinking of the marginal zone, regions where the majority of splenic macrophages reside (Borges da Silva et al., 2015). Our results bear resemblance to the impairment of *B. microti*-conferred cross-protection against fatal *B. rodhaini* following macrophage depletion (Li et al., 2012). Given the decisive role of macrophages in resistance to babesiosis (Aguilar-Delfin et al., 2003; Terkawi et al., 2015; Djokic et al., 2018a) and non-lethal murine malaria (Terkawi et al., 2016), it appears plausible that a macrophage-based immune mechanism induced by *B. microti* may carry on its protective effect against a subsequent *P. chabaudi* challenge. One immune function of macrophages is the production of TNF-α (Clark et al., 1987; Stevenson et al., 1989), which causes the release of ROS and NO, soluble mediators that induce intraerythrocytic degeneration of *Plasmodium* and *Babesia* (Clark and Hunt, 1983; Johnson et al., 1996; Chua et al., 2013). However, protected mice in our experiments did not exhibit higher TNF-α levels after challenge infection. Interestingly, a novel mechanism of intraerythrocytic killing of *B. microti*, which is independent of iNOS has recently been described (Skariah et al., 2017). This mechanism is associated with increased production of IL-10 and does not require adaptive immunity, which is in agreement with our observations of cross-protection in BALB/c and SCID mice, respectively. If non-specific in nature, this yet to-be elucidated apparatus could be responsible for the cross-protection described in our study. By virtue of their Th2-biased nature, BALB/c macrophages have impaired NO production in response to bacteria, resulting in a lack of effector molecules for indirect killing (Watanabe et al., 2004). However, *B. microti* infection triggers early-stage macrophage activation, essential in controlling parasite replication (Terkawi et al., 2015). We thus hypothesize that effective macrophage activation caused by and sustained throughout a primary *B. microti* infection can both mediate the removal of pRBCs (which would explain the low levels of *P. chabaudi*-induced parasitemia in protected mice), while also regulating the response of other immune effector cells to the *P. chabaudi* challenge. This could help mice successfully control the deadly first wave of parasitemia, while also avoiding the immunopathology associated with excessive inflammatory responses to malaria.

This study has a number of limitations. The high *B. microti* infectious dose used in our model does not accurately reflect the dose deposited by tick vectors during natural infection of wild rodents. Nevertheless, similar doses have been frequently used in past studies of murine *B. microti* infection (Igarashi et al., 1999; Li et al., 2012; Terkawi et al., 2015; Yi et al., 2018). The kinetics of infection, including the course of parasitemia and IgG and IgM responses appear similar between higher and lower *B. microti* doses (Bakkour et al., 2018). We therefore have reasons to believe a lower infectious dose would have yielded similar results in our model. It is, however, plausible that artificial inoculation may eliminate the important variable of tick salivary molecules enhancing the establishment of a natural infection (Knapp and Rice, 2015). Another point is that only a single strain of each parasite was used in our study. Yet, as different pathogen genotypes can lead to mixed immunological responses, dissimilar levels of disease manifestation reported in *Babesia*-*Plasmodium* co-infection studies could be due to the different genetic variants involved. Regrettably, none of the human co-infection reports reached pathogen identification beyond the species level, and so the impact of the pathogen genetic background in ensuing pathogenicity remains unknown. The above should be taken into consideration before attempting direct comparisons between cases. Furthermore, as the protocol we employed for depletion of macrophages/monocytes generally leads to systemic exhaustion, no specific macrophage population (like splenic or blood) can be associated with the mechanism of cross-protection. Additional experiments are required to decipher the role of different tissue-specific macrophage populations in cross-protection. Targeting splenic macrophages would be of special interest, as recent studies show that splenic macrophages increase in number during acute *B. microti* infection and facilitate clearance of infected erythrocytes (Djokic et al., 2018a,b, 2019). Further work will also be required to dissect the role of different macrophage subtypes, like M1 and M2, in crafting the delicate balance between pro- and anti-inflammatory cytokines that we hypothesize to facilitate cross-protection. Toward that end, examining the cytokine response in macrophage-depleted mice will be vital. Lastly, as production of soluble mediators of non-specific parasite killing, such as ROS and NO, was not monitored in the present study, the exact molecules involved in the cross-protective mechanism remain unknown.

As discussed above, multiple studies have established the presence of heterologous immunity between *Babesia* and *Plasmodium* in primates, suggesting that such an interaction may also occur in our species. In that case, it would be interesting to explore whether co-infection in humans could have the same protective effect against malaria. Historically, vaccine development efforts against malaria have been strongly focused on adaptive immunity, despite clinical studies in humans indicating that innate immune responses help control malaria and can influence the level of susceptibility to the disease (Kwiatkowski, 2000; Molineaux et al., 2002). Furthermore, innate immune system activation can boost the immunogenicity of recombinant malaria antigens (Su et al., 2003; Stevenson and Riley, 2004). Given the common characteristics of *Babesia* and *Plasmodium*, the encouraging phenomenon of innate immunity-based cross-protection shown here, and the ongoing successful use of *Babesia* as an attenuated vaccine in veterinary applications (Shkap et al., 2007), it is worth considering the potential of genetically altered *Babesia* as a proxy for delivering live malaria vaccines to humans.

In light of climate change and the concerning implications regarding the geographic range and incidence of arthropod-borne infectious diseases (McMichael et al., 2006; Cotter et al., 2013), human cases of *Plasmodium* and *Babesia* co-infection are expected to increase in the near future. It is thus worthwhile to understand the way these parasites interact in the host and affect the outcome of co-infection. In the present study we demonstrated that *B. microti* infection confers broad innate immunity-based cross-protection against *P. chabaudi* in mice, with macrophages being a key contributor. Further research is needed to elucidate the malaria-suppressing effects of babesiosis, with an aim toward inventing novel tools for malaria control.

## Data Availability Statement

The raw data supporting the conclusions of this article will be made available by the authors, without undue reservation, to any qualified researcher.

## Ethics Statement

The animal study was reviewed and approved by Research Ethics Review Committee of the Obihiro University of Agriculture and Veterinary Medicine (approval number 28–99).

## Author Contributions

AE and XX conceived and designed the study. AE executed the mouse experiments, laboratory, and flow cytometry assays, analyzed and interpreted the data, and prepared the manuscript. EG assisted with the mouse experiments, laboratory and flow cytometry assays, and helped with data analysis. GW assisted with the mouse experiments and laboratory assays. YN, HS, and II provided valuable suggestions throughout the study. DK performed the histopathological examination. MT helped design and KU supervised the flow cytometry assays. AK provided the *Plasmodium* parasite stocks. XX revised the manuscript. ML, AR, HG, YG, S-HL, JL, and PM proof-read the manuscript. All authors have reviewed and approved the final manuscript.

## Conflict of Interest

The authors declare that the research was conducted in the absence of any commercial or financial relationships that could be construed as a potential conflict of interest.

## References

[B1] Abdel T. N. S. Abdoun A. M. El M. T. (1998). Babesia parasites described from patients bled for malaria. Saudi Med. J. 19, 179–181. 27701582

[B2] Aguilar-Delfin I. Homer M. J. Wettstein P. J. Persing D. H. (2001). Innate resistance to *Babesia* infection is influenced by genetic background and gender. Infect. Immun. 69, 7955–7958. 10.1128/IAI.69.12.7955-7958.2001 11705985 PMC98899

[B3] Aguilar-Delfin I. Wettstein P. J. Persing D. H. (2003). Resistance to acute babesiosis is associated with interleukin-12-and gamma interferon-mediated responses and requires macrophages and natural killer cells. Infect. Immun. 71, 2002–2008. 10.1128/IAI.71.4.2002-2008.2003 12654819 PMC152030

[B4] Ahn M. H. (2010). Imported parasitic diseases in Korea. Infect. Chemother. 42, 271–279. 10.3947/ic.2010.42.5.271

[B5] Allred D. R. (2003). Babesiosis: persistence in the face of adversity. Trends Parasitol. 19, 51–55. 10.1016/S1471-4922(02)00065-X 12586467

[B6] Allred D. R. Al-Khedery B. (2004). Antigenic variation and cytoadhesion in *Babesia bovis* and *Plasmodium falciparum*: different logics achieve the same goal. Mol. Biochem. Parasitol. 134, 27–35. 10.1016/j.molbiopara.2003.09.012 14747140

[B7] Artavanis-Tsakonas K. Riley E. M. (2002). Innate immune response to malaria: rapid induction of IFN-γ from human NK cells by live Plasmodium falciparum-infected erythrocytes. J. Immunol. 169, 2956–2963. 10.4049/jimmunol.169.6.2956 12218109

[B8] Bakkour S. Chafets D. M. Wen L. Muench M. O. Telford S. R. 3rd Erwin, J. L.. (2018). Minimal infectious dose and dynamics of Babesia microti parasitemia in a murine model. Transfusion 58, 2903–2910. 10.1111/trf.14889 30264498

[B9] Barton E. S. White D. W. Cathelyn J. S. Brett-McClellan K. A. Engle M. Diamond M. S.. (2007). Herpesvirus latency confers symbiotic protection from bacterial infection. Nature 447:326. 10.1038/nature05762 17507983

[B10] Bhanot P. and Parveen, N. (2019). Investigating disease severity in an animal model of concurrent babesiosis and Lyme disease. Int. J. Parasitol. 49, 145–151. 10.1016/j.ijpara.2018.06.006 30367867 PMC6399035

[B11] Bloch E. M. Kumar S. Krause P. J. (2019). Persistence of *Babesia microti* infection in humans. Pathogens 8:102. 10.3390/pathogens8030102 31319461 PMC6789900

[B12] Borges da Silva H. Fonseca R. Pereira R. M. Cassado A. D. A. Álvarez J. M. D'Império Lima M. R. (2015). Splenic macrophage subsets and their function during blood-borne infections. Front. Immunol. 6:480. 10.3389/fimmu.2015.00480 26441984 PMC4585205

[B13] Bruce M. C. Day K. P. (2002). Cross-species regulation of malaria parasitaemia in the human host. Curr. Opin. Microbiol. 5, 431–437. 10.1016/S1369-5274(02)00348-X 12160865

[B14] Buffet P. A. Safeukui I. Deplaine G. Brousse V. Prendki V. Thellier M.. (2011). The pathogenesis of *Plasmodium falciparum* malaria in humans: insights from splenic physiology. Blood J. Am. Soc. Hematol. 117, 381–392. 10.1182/blood-2010-04-202911 20852127 PMC3031473

[B15] Bush J. B. Isaäcson M. Mohamed A. S. Potgieter F. T. De Waal D. T. (1990). Human babesiosis-a preliminary report of 2 suspected cases in southern Africa. S Afr. Med. J. 78:699. 2251622

[B16] Chua C. L. L. Brown G. Hamilton J. A. Rogerson S. Boeuf P. (2013). Monocytes and macrophages in malaria: protection or pathology? Trends Parasitol. 29, 26–34. 10.1016/j.pt.2012.10.002 23142189

[B17] Clark I. A. Cowden W. B. Butcher G. A. Hunt N. H. (1987). Possible roles of tumor necrosis factor in the pathology of malaria. Am. J. Pathol. 129:192. 3661678 PMC1899696

[B18] Clark I. A. Hunt N. H. (1983). Evidence for reactive oxygen intermediates causing hemolysis and parasite death in malaria. Infect. Immun. 39, 1–6. 10.1128/IAI.39.1.1-6.1983 6822409 PMC347899

[B19] Clawson M. L. Paciorkowski N. Rajan T. V. La Vake C. Pope C. La Vake M. (2002). Cellular immunity, but not gamma interferon, is essential for resolution of *Babesia microti* infection in BALB/c mice. Infect. Immun. 70, 5304–5306. 10.1128/IAI.70.9.5304-5306.2002 12183588 PMC128260

[B20] Coleman J. L. LeVine D. Thill C. Kuhlow C. Benach J. L. (2005). *Babesia microti* and *Borrelia burgdorferi* follow independent courses of infection in mice. J. Infect. Dis. 192, 1634–1641. 10.1086/496891 16206079

[B21] Collins W. E. Jeffery G. M. (1999). A retrospective examination of sporozoite-and trophozoite-induced infections with Plasmodium falciparum in patients previously infected with heterologous species of Plasmodium: effect on development of parasitologic and clinical immunity. Am. J. Trop. Med. Hyg. 61(Suppl.1), 36–43. 10.4269/tropmed.1999.61-036 10432043

[B22] Cotter C. Sturrock H. J. Hsiang M. S. Liu J. Phillips A. A. Hwang J.. (2013). The changing epidemiology of malaria elimination: new strategies for new challenges. Lance 382, 900–911. 10.1016/S0140-6736(13)60310-4 23594387 PMC10583787

[B23] Couper K. N. Blount D. G. Hafalla J. C. van Rooijen N. de Souza J. B. Riley E. M. (2007). Macrophage-mediated but gamma interferon-independent innate immune responses control the primary wave of *Plasmodium yoelii* parasitemia. Infect. Immun. 75, 5806–5818. 10.1128/IAI.01005-07 17923512 PMC2168355

[B24] Couper K. N. Blount D. G. Riley E. M. (2008). IL-10: the master regulator of immunity to infection. J. Immunol. 180, 5771–5777. 10.4049/jimmunol.180.9.5771 18424693

[B25] Cox F. E. G. (1972). Protective heterologous immunity between *Plasmodium atheruri* and other *Plasmodium* spp. and *Babesia* spp. in mice. Parasitology 65, 379–387. 10.1017/S0031182000044000 4629791

[B26] Cox F. E. G. (1978). Heterologous immunity between piroplasms and malaria parasites: the simultaneous elimination of *Plasmodium vinckei* and *Babesia microti* from the blood of doubly infected mice. Parasitology 76, 55–60. 10.1017/S0031182000047387 622306

[B27] Cross C. E. Langhorne J. (1998). *Plasmodium chabaudi* chabaudi (AS): inflammatory cytokines and pathology in an erythrocytic-stage infection in mice. Exp. Parasitol. 90, 220–229. 10.1006/expr.1998.4335 9806866

[B28] Djokic V. Akoolo L. Parveen N. (2018a). *Babesia microti* infection changes host spleen architecture and is cleared by a Th1 immune response. Front. Microbiol. 9:85. 10.3389/fmicb.2018.00085 29445365 PMC5797759

[B29] Djokic V. Akoolo L. Primus S. Schlachter S. Kelly K. Bhanot P.. (2019). Protozoan parasite *Babesia microti* subverts adaptive immunity and enhances Lyme disease severity. Front. Microbiol. 10:1596. 10.3389/fmicb.2019.01596 31354683 PMC6635642

[B30] Djokic V. Primus S. Akoolo L. Chakraborti M. Parveen N. (2018b). Age-related differential stimulation of immune response by *Babesia microti* and *Borrelia burgdorferi* during acute phase of infection affects disease severity. Front. Immunol. 9:2891. 10.3389/fimmu.2018.02891 30619263 PMC6300717

[B31] Doolan D. L. Dobaño C. Baird J. K. (2009). Acquired immunity to malaria. Clin. Microbiol. Rev. 22, 13–36. 10.1128/CMR.00025-08 19136431 PMC2620631

[B32] Engwerda C. R. Beattie L. Amante F. H. (2005). The importance of the spleen in malaria. Trends Parasitol. 21, 75–80. 10.1016/j.pt.2004.11.008 15664530

[B33] Engwerda C. R. Mynott T. L. Sawhney S. De Souza J. B. Bickle Q. D. Kaye P. M. (2002). Locally up-regulated lymphotoxin α, not systemic tumor necrosis factor α, is the principle mediator of murine cerebral malaria. J. Exp. Med. 195, 1371–1377. 10.1084/jem.20020128 12021316 PMC2193758

[B34] Gabrielli S. Bellina L. Milardi G. L. Katende B. K. Totino V. Fullin V.. (2016). Malaria in children of Tshimbulu (Western Kasai, Democratic Republic of the Congo): epidemiological data and accuracy of diagnostic assays applied in a limited resource setting. Malaria J. 15:81. 10.1186/s12936-016-1142-8 26864461 PMC4750168

[B35] Good M. F. Doolan D. L. (1999). Immune effector mechanisms in malaria. Curr. Opin. Immunol. 11, 412–419. 10.1016/S0952-7915(99)80069-7 10448141

[B36] Homer M. J. Aguilar-Delfin I. Telford S. R. Krause P. J. Persing D. H. (2000). Babesiosis. Clin. Microbiol. Rev. 13, 451–469. 10.1128/CMR.13.3.451 10885987 PMC88943

[B37] Igarashi I. Suzuki R. Waki S. Tagawa Y. I. Seng S. Tum S.. (1999). Roles of CD4+ T cells and gamma interferon in protective immunity against babesia microtiinfection in mice. Infect. Immun. 67, 4143–4148. 10.1128/IAI.67.8.4143-4148.1999 10417185 PMC96718

[B38] Jeong Y. I. Hong S. H. Cho S. H. Lee W. J. Lee S. E. (2012). Induction of IL-10-producing CD1d^high^CD5+ regulatory B cells following *Babesia microti*-infection. PLoS ONE 7:e46553. 10.1371/journal.pone.0046553 23071588 PMC3465325

[B39] Johnson W. C. Cluff C. W. Goff W. L. Wyatt C. R. (1996). Reactive oxygen and nitrogen intermediates and products from polyamine degradation are Babesiacidal *in vitro*. Ann. N. Y. Acad. Sci. 791, 136–147. 10.1111/j.1749-6632.1996.tb53520.x 8784495

[B40] Jones K. E. Patel N. G. Levy M. A. Storeygard A. Balk D. Gittleman J. L.. (2008). Global trends in emerging infectious diseases. Nature 451:990. 10.1038/nature06536 18288193 PMC5960580

[B41] Knapp K. L. Rice N. A. (2015). Human coinfection with *Borrelia burgdorferi* and *Babesia microti* in the United States. J. Parasitol. Res. 2015:587131. 10.1155/2015/587131 26697208 PMC4677215

[B42] Kwiatkowski D. (2000). Genetic susceptibility to malaria getting complex. Curr. Opin. Genet. Dev. 10, 320–324. 10.1016/S0959-437X(00)00087-3 10826994

[B43] Langhorne J. Gillard S. Simon B. Slade S. Eichmann K. (1989). Frequencies of CD4+ T cells reactive with *Plasmodium chabaudi* chabaudi: distinct response kinetics for cells with Th1 and Th2 characteristics during infection. Int. Immunol. 1, 416–424. 10.1093/intimm/1.4.416 2535135

[B44] Langhorne J. Ndungu F. M. Sponaas A. M. Marsh K. (2008). Immunity to malaria: more questions than answers. Nat. Immunol. 9:725. 10.1038/ni.f.205 18563083

[B45] Langhorne J. Quin S. J. Sanni L. A. (2002). Mouse models of blood-stage malaria infections: immune responses and cytokines involved in protection and pathology. Chem. Immunol. 80, 204–228. 10.1159/000058845 12058640

[B46] Leisewitz A. L. Rockett K. A. Gumede B. Jones M. Urban B. Kwiatkowski D. P. (2004). Response of the splenic dendritic cell population to malaria infection. Infect. Immun. 72, 4233–4239. 10.1128/IAI.72.7.4233-4239.2004 15213168 PMC427429

[B47] Li C. Corraliza I. Langhorne J. (1999). A defect in interleukin-10 leads to enhanced malarial disease in *Plasmodium chabaudi* chabaudi infection in mice. Infect. Immun. 67, 4435–4442. 10.1128/IAI.67.9.4435-4442.1999 10456884 PMC96762

[B48] Li C. Sanni L. A. Omer F. Riley E. Langhorne J. (2003). Pathology of *Plasmodium chabaudi* chabaudi infection and mortality in interleukin-10-deficient mice are ameliorated by anti-tumor necrosis factor alpha and exacerbated by anti-transforming growth factor β antibodies. Infect. Immun. 71, 4850–4856. 10.1128/IAI.71.9.4850-4856.2003 12933825 PMC187303

[B49] Li Y. Terkawi M. A. Nishikawa Y. Aboge G. O. Luo Y. Ooka H.. (2012). Macrophages are critical for cross-protective immunity conferred by *Babesia microti* against *Babesia rodhaini* infection in mice. Infect. Immun. 80, 311–320. 10.1128/IAI.05900-11 22064713 PMC3255693

[B50] Looareesuwan S. Ho M. Wattanagoon Y. White N. J. Warrell D. A. Bunnag D.. (1987). Dynamic alteration in splenic function during acute falciparum malaria. N. Engl. J. Med. 317, 675–679. 10.1056/NEJM198709103171105 3306376

[B51] Loutan L. Rossier J. Zufferey G. Cuenod D. Hatz C. Marti H. P.. (1993). Imported babesiosis diagnosed as malaria. Lancet 342:749. 10.1016/0140-6736(93)91744-7 8103861

[B52] Marsh K. Kinyanjui S. (2006). Immune effector mechanisms in malaria. Parasite Immunol. 28, 51–60. 10.1111/j.1365-3024.2006.00808.x 16438676

[B53] McMichael A. J. Woodruff R. E. Hales S. (2006). Climate change and human health: present and future risks. Lancet 367, 859–869. 10.1016/S0140-6736(06)68079-3 16530580

[B54] Meding S. J. Langhorne J. (1991). CD4+ T cells and B cells are necessary for the transfer of protective immunity to *Plasmodium chabaudi* chabaudi. Eur. J. Immunol. 21, 1433–1438. 10.1002/eji.1830210616 1675172

[B55] Mohan K. Moulin P. Stevenson M. M. (1997). Natural killer cell cytokine production, not cytotoxicity, contributes to resistance against blood-stage *Plasmodium chabaudi* AS infection. J. Immunol. 159, 4990–4998. 9366426

[B56] Molineaux L. Träuble M. Collins W. E. Jeffery G. M. Dietz K. (2002). Malaria therapy reinoculation data suggest individual variation of an innate immune response and independent acquisition of antiparasitic and antitoxic immunities. Trans. R Soc. Trop. Med. Hyg. 96, 205–209. 10.1016/S0035-9203(02)90308-1 12055817

[B57] Moro M. H. Zegarra-Moro O. L. Bjornsson J. Hofmeister E. K. Bruinsma E. Germer J. J.. (2002). Increased arthritis severity in mice coinfected with *Borrelia burgdorferi* and *Babesia microti*. J. Infect. Dis. 186, 428–431. 10.1086/341452 12134242

[B58] Na Y. J. Chai J. Y. Jung B. K. Lee H. J. Song J. Y. Je J. H.. (2014). An imported case of severe falciparum malaria with prolonged hemolytic anemia clinically mimicking a coinfection with babesiosis. Korean J. Parasitol. 52:667. 10.3347/kjp.2014.52.6.667 25548419 PMC4277030

[B59] Niikura M. Kamiya S. Kita K. Kobayashi F. (2008). Coinfection with nonlethal murine malaria parasites suppresses pathogenesis caused by *Plasmodium berghei* NK65. J. Immunol. 180, 6877–6884. 10.4049/jimmunol.180.10.6877 18453608

[B60] Page K. R. Scott A. L. Manabe Y. C. (2006). The expanding realm of heterologous immunity: friend or foe? Cell Microbiol. 8, 185–196. 10.1111/j.1462-5822.2005.00653.x 16441430

[B61] Pawelczyk A. Bajer A. Behnke J. M. Gilbert F. S. Sinski E. (2004). Factors affecting the component community structure of haemoparasites in common voles (*Microtus arvalis*) from the Mazury Lake District region of Poland. Parasitol. Res. 92, 270–284. 10.1007/s00436-003-1040-1 14714180

[B62] Pedersen A. B. Fenton A. (2007). Emphasizing the ecology in parasite community ecology. Trends Ecol. Evol. 22, 133–139. 10.1016/j.tree.2006.11.005 17137676

[B63] Redpath S. A. Fonseca N. M. Perona-Wright G. (2014). Protection and pathology during parasite infection: IL-10 strikes the balance. Parasite Immunol. 36, 233–252. 10.1111/pim.12113 24666543

[B64] Riley E. M. (1999). Is T-cell priming required for initiation of pathology in malaria infections? Immunol. Today 20, 228–233. 10.1016/S0167-5699(99)01456-5 10322302

[B65] Scott P. Pearce E. Cheever A. W. Coffman R. L. Sher A. (1989). Role of cytokines and CD4+ T-cell subsets in the regulation of parasite immunity and disease. Immunol. Rev. 112, 161–182. 10.1111/j.1600-065X.1989.tb00557.x 2575073

[B66] Seixas E. Ostler D. (2005). *Plasmodium chabaudi* chabaudi (AS): differential cellular responses to infection in resistant and susceptible mice. Exp. Parasitol. 110, 394–405. 10.1016/j.exppara.2005.03.024 15953500

[B67] Selin L. K. Brehm M. A. Naumov Y. N. Cornberg M. Kim S. K. Clute S. C.. (2006). Memory of mice and men: CD8+ T-cell cross-reactivity and heterologous immunity. Immunol. Rev. 211, 164–181. 10.1111/j.0105-2896.2006.00394.x 16824126 PMC7165519

[B68] Selin L. K. Varga S. M. Wong I. C. Welsh R. M. (1998). Protective heterologous antiviral immunity and enhanced immunopathogenesis mediated by memory T cell populations. J. Exp. Med. 188, 1705–1715. 10.1084/jem.188.9.1705 9802982 PMC2212518

[B69] Shkap V. de Vos A. J. Zweygarth E. Jongejan F. (2007). Attenuated vaccines for tropical theileriosis, babesiosis and heartwater: the continuing necessity. Trends Parasitol. 23, 420–426. 10.1016/j.pt.2007.07.003 17656155

[B70] Skariah S. Arnaboldi P. Dattwyler R. J. Sultan A. A. Gaylets C. Walwyn O.. (2017). Elimination of *Babesia microti* is dependent on intraerythrocytic killing and CD4+ T cells. J. Immunol. 199, 633–642. 10.4049/jimmunol.1601193 28607116 PMC5557026

[B71] Springer A. Fichtel C. Calvignac-Spencer S. Leendertz F. H. Kappeler P. M. (2015). Hemoparasites in a wild primate: infection patterns suggest interaction of *Plasmodium* and *Babesia* in a lemur species. Int. J. Parasitol. Parasites Wildlife 4, 385–395. 10.1016/j.ijppaw.2015.10.006 26767166 PMC4683568

[B72] Stephens R. Albano F. R. Quin S. Pascal B. J. Harrison V. Stockinger B.. (2005). Malaria-specific transgenic CD4+ T cells protect immunodeficient mice from lethal infection and demonstrate requirement for a protective threshold of antibody production for parasite clearance. Blood 106, 1676–1684. 10.1182/blood-2004-10-4047 15890689

[B73] Stephens R. Culleton R. L. Lamb T. J. (2012). The contribution of *Plasmodium chabaudi* to our understanding of malaria. Trends Parasitol. 28, 73–82. 10.1016/j.pt.2011.10.006 22100995 PMC4040349

[B74] Stevenson M. M. Ghadirian E. Phillips N. C. Rae D. Podoba J. E. (1989). Role of mononuclear phagocytes in elimination of *Plasmodium chabaudi* AS infection. Parasite Immunol. 11, 529–544. 10.1111/j.1365-3024.1989.tb00687.x 2555763

[B75] Stevenson M. M. Lyanga J. J. Skamene E. (1982). Murine malaria: genetic control of resistance to *Plasmodium chabaudi*. Infect. Immun. 38, 80–88. 10.1128/IAI.38.1.80-88.1982 7141699 PMC347700

[B76] Stevenson M. M. Riley E. M. (2004). Innate immunity to malaria. Nat. Rev. Immunol. 4:169. 10.1038/nri1311 15039754

[B77] Stevenson M. M. Tam M. F. Wolf S. F. Sher A. (1995). IL-12-induced protection against blood-stage *Plasmodium chabaudi* AS requires IFN-gamma and TNF-alpha and occurs via a nitric oxide-dependent mechanism. J. Immunol. 155, 2545–2556. 7650384

[B78] Su Z. Stevenson M. M. (2000). Central role of endogenous gamma interferon in protective immunity against blood-stage *Plasmodium chabaudi* AS infection. Infect. Immun. 68, 4399–4406. 10.1128/IAI.68.8.4399-4406.2000 10899836 PMC98333

[B79] Su Z. Tam M. F. Jankovic D. Stevenson M. M. (2003). Vaccination with novel immunostimulatory adjuvants against blood-stage malaria in mice. Infect. Immun. 71, 5178–5187. 10.1128/IAI.71.9.5178-5187.2003 12933862 PMC187300

[B80] Tanaka S. Nishimura M. Ihara F. Yamagishi J. Suzuki Y. Nishikawa Y. (2013). Transcriptome analysis of mouse brain infected with *Toxoplasma gondii*. Infect. Immun. 81, 3609–3619. 10.1128/IAI.00439-13 23856619 PMC3811780

[B81] Telfer S. Birtles R. Bennett M. Lambin X. Paterson S. Begon M. (2008). Parasite interactions in natural populations: insights from longitudinal data. Parasitology 135, 767–781. 10.1017/S0031182008000395 18474121 PMC2952918

[B82] Terkawi M. A. Cao S. Herbas M. S. Nishimura M. Li Y. Moumouni P. F. A.. (2015). Macrophages are the determinant of resistance to and outcome of nonlethal *Babesia microti* infection in mice. Infect. Immun. 83, 8–16. 10.1128/IAI.02128-14 25312951 PMC4288901

[B83] Terkawi M. A. Nishimura M. Furuoka H. Nishikawa Y. (2016). Depletion of phagocytic cells during nonlethal *Plasmodium yoelii* infection causes severe malaria characterized by acute renal failure in mice. Infect. Immun. 84, 845–855. 10.1128/IAI.01005-15 26755155 PMC4771365

[B84] Todryk S. M. Bejon P. Mwangi T. Plebanski M. Urban B. Marsh K. (2008). Correlation of memory T cell responses against TRAP with protection from clinical malaria, and CD4+ CD25high T cells with susceptibility in Kenyans. PLoS ONE 3:e2027 10.1371/journal.pone.0002027 18446217 PMC2323567

[B85] Trinchieri G. (2007). Interleukin-10 production by effector T cells: Th1 cells show self control. J. Exp. Med. 204, 239–243. 10.1084/jem.20070104 17296790 PMC2118719

[B86] van Duivenvoorde L. M. Voorberg-van der Wel A. van der Werff N. M. Braskamp G. Remarque E. J. Kondova I.. (2010). Suppression of *Plasmodium cynomolgi* in rhesus macaques by coinfection with *Babesia microti*. Infect. Immun. 78, 1032–1039. 10.1128/IAI.00921-09 20048045 PMC2825946

[B87] Vannier E. Krause P. J. (2012). Human babesiosis. N. Engl. J. Med. 366, 2397–2407. 10.1056/NEJMra1202018 22716978

[B88] Vermeil C. Menut J. Miegeville M. Cruziat J. Julienne F. Morin O.. (1983). Babesiasis, pediatric malaria: does confusion exist in Africa? Bull. Soc. Pathol. Exot. 76, 797–804. 6368023

[B89] von der Weid T. Honarvar N. Langhorne J. (1996). Gene-targeted mice lacking B cells are unable to eliminate a blood stage malaria infection. J. Immunol. 156, 2510–2516. 8786312

[B90] Watanabe H. Numata K. Ito T. Takagi K. Matsukawa A. (2004). Innate immune response in Th1-and Th2-dominant mouse strains. Shock 22, 460–466. 10.1097/01.shk.0000142249.08135.e9 15489639

[B91] Waterfall M. Black A. Riley E. (1998). γδ+ T cells preferentially respond to live rather than killed malaria parasites. Infect. Immun. 66, 2393–2398. 10.1128/IAI.66.5.2393-2398.1998 9573139 PMC108213

[B92] Wel A. V. Kocken C. H. Zeeman A. M. Thomas A. W. (2008). Detection of new *Babesia microti*-like parasites in a rhesus monkey (*Macaca mulatta*) with a suppressed *Plasmodium cynomolgi* infection. Am. J. Trop. Med. Hyg. 78, 643–645. 10.4269/ajtmh.2008.78.643 18385363

[B93] Welc-Faleciak R. Bajer A. Bednarska M. Paziewska A. Sinski E. (2007). Long term monitoring of *Babesia microti* infection in BALB-c mice using nested PCR. Ann. Agr. Env. Med. 14, 287–90. 18247466

[B94] Welsh R. M. Che J. W. Brehm M. A. Selin L. K. (2010). Heterologous immunity between viruses. Immunol. Rev. 235, 244–266. 10.1111/j.0105-2896.2010.00897.x 20536568 PMC2917921

[B95] World Health Organization. (2016). World Malaria Report 2015. Geneva: World Health Organization.

[B96] Wu Y. Wang Q. H. Zheng L. Feng H. Liu J. Ma S. H.. (2007). *Plasmodium yoelii*: distinct CD4+ CD25+ regulatory T cell responses during the early stages of infection in susceptible and resistant mice. Exp. Parasitol. 115, 301–304. 10.1016/j.exppara.2006.09.015 17084842

[B97] Wunderlich F. Mossmann H. Helwig M. Schillinger G. (1988). Resistance to *Plasmodium chabaudi* in B10 mice: influence of the H-2 complex and testosterone. Infect. Immun. 56, 2400–2406. 10.1128/IAI.56.9.2400-2406.1988 3410544 PMC259579

[B98] Wyler D. J. Quinn T. C. Chen L. T. (1981). Relationship of alterations in splenic clearance function and microcirculation to host defense in acute rodent malaria. J. Clin. Invest. 67, 1400–1404. 10.1172/JCI110168 7014635 PMC370706

[B99] Yadava A. Kumar S. Dvorak J. A. Milon G. Miller L. H. (1996). Trafficking of *Plasmodium chabaudi* adami-infected erythrocytes within the mouse spleen. Proc. Natl Acad. Sci. U. S. A. 93, 4595–4599. 10.1073/pnas.93.10.4595 8643449 PMC39322

[B100] Yi W. Bao W. Rodriguez M. Liu Y. Singh M. Ramlall V.. (2018). Robust adaptive immune response against *Babesia microti* infection marked by low parasitemia in a murine model of sickle cell disease. Blood Adv. 2, 3462–3478. 10.1182/bloodadvances.2018026468 30518538 PMC6290097

[B101] Yoshimoto T. Takahama Y. Wang C. R. Yoneto T. Waki S. Nariuchi H. (1998). A pathogenic role of IL-12 in blood-stage murine malaria lethal strain *Plasmodium berghei* NK65 infection. J. Immunol. 160, 5500–5505. 9605153

[B102] Zhou X. Li S. G. Chen S. B. Wang J. Z. Xu B. Zhou H. J.. (2013). Co-infections with *Babesia microti* and *Plasmodium* parasites along the China-Myanmar border. Infect. Dis. Poverty 2:24. 10.1186/2049-9957-2-24 24090043 PMC3819642

[B103] Zivkovic D. Speksnijder J. E. Kuil H. Seinen W. (1984). Immunity to *Babesia* in mice II. cross protection between various *Babesia* and *Plasmodium* species and its relevance to the nature of *Babesia* immunity. Vet. Immunol. Immunopathol. 5, 359–368. 10.1016/0165-2427(84)90004-7 6730311

