# Supplementary material for: Babesia microti Confers Macrophage-Based Cross-Protective Immunity Against Murine Malaria
Source: Front Cell Infect Microbiol. 2020 Apr 29;10:193. doi: 10.3389/fcimb.2020.00193 (PMC7200999; doi:10.3389/fcimb.2020.00193)
Supplement: Supplementary file 1 [file Data_Sheet_1.docx]

Supplementary Material


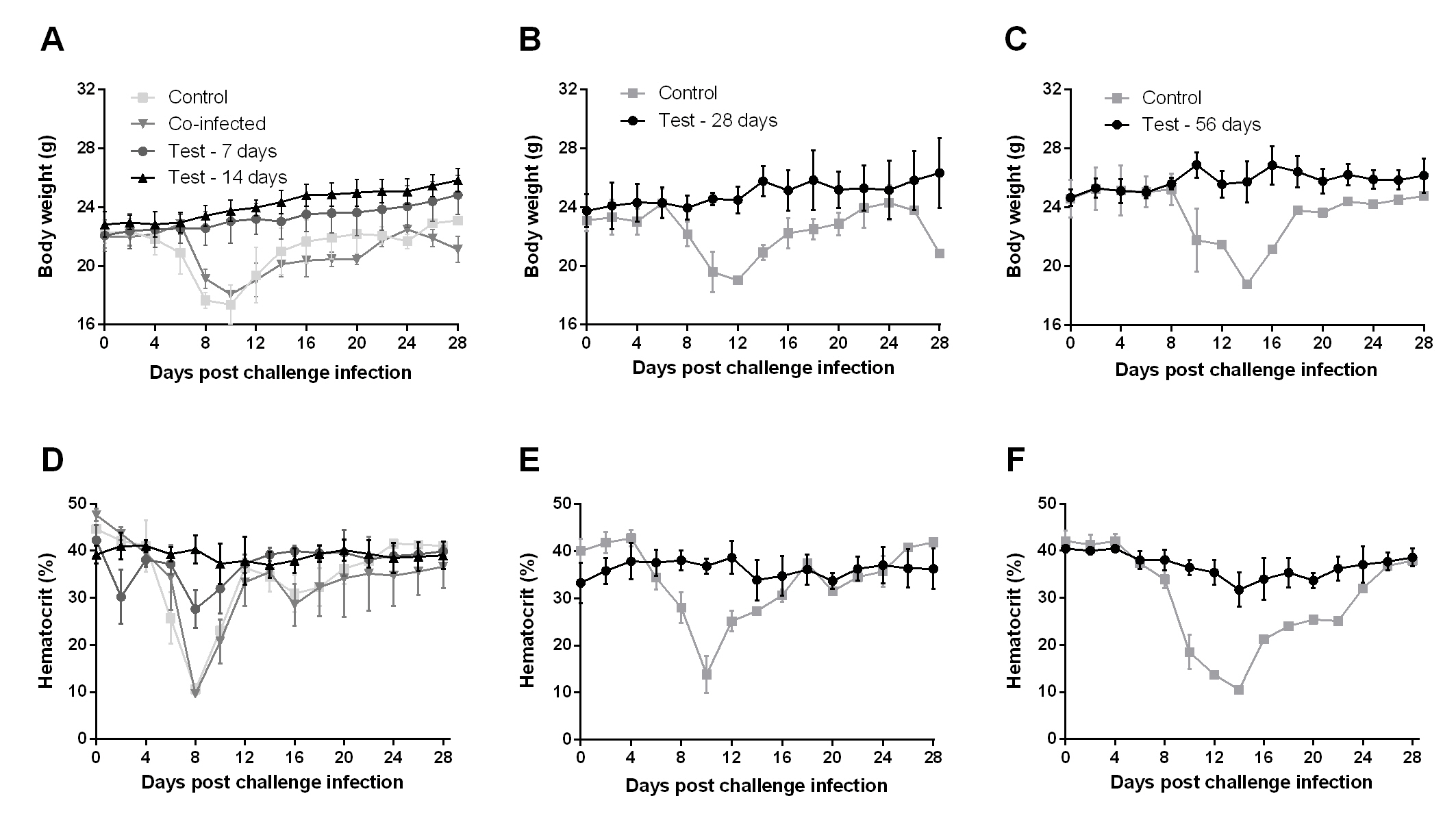


**Supplementary Figure 1.** **Course of *P. chabaudi* challenge infection in BALB/c mice undergoing different stages of primary *B. microti* infection**. Test BALB/c mice were initially infected with *B. microti* and then challenge-infected with *P. chabaudi* at different time points (on days 0, 7, 14, 28, or 56) post primary infection. Control mice received *P. chabaudi* alone. Body weight (**A**, **B**, and **C**) and hematocrit (**D**, **E**, and **F**) of control and test mice are presented. Results are expressed as mean values ± the standard deviation (SD) of five or six mice. Mean values are calculated from individual values taken from all surviving mice at each specific time point. Experiment shown is representative of three independent experiments. Experiments of challenge infection on days 0, 7, and 14 (**A, D**) belonged to a single cohort and as such share the same control group.


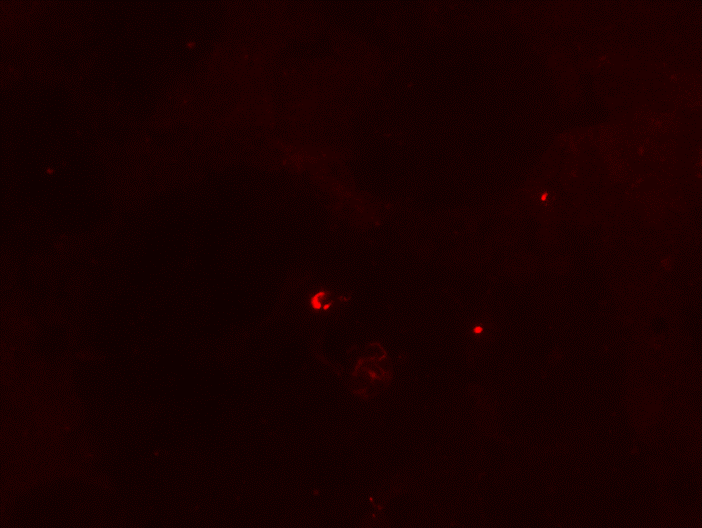

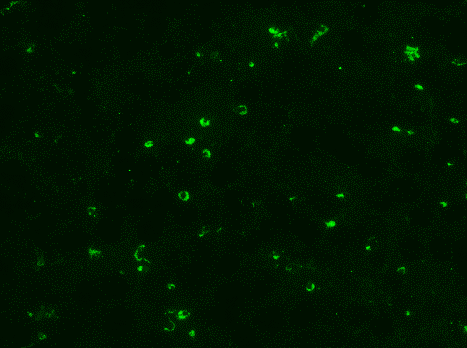

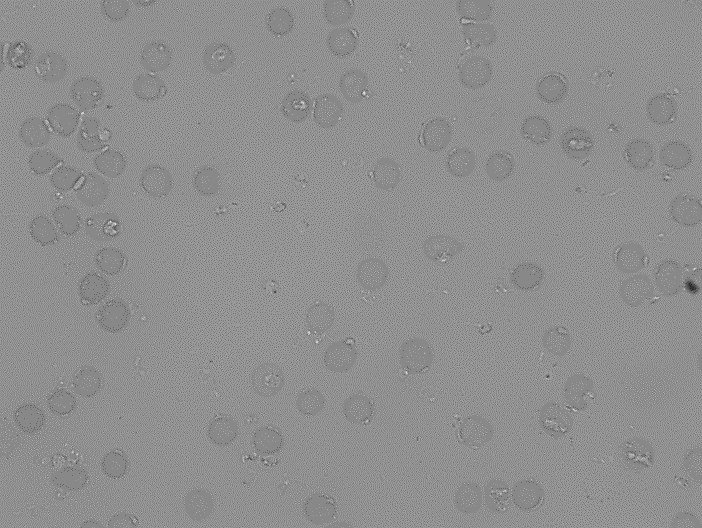

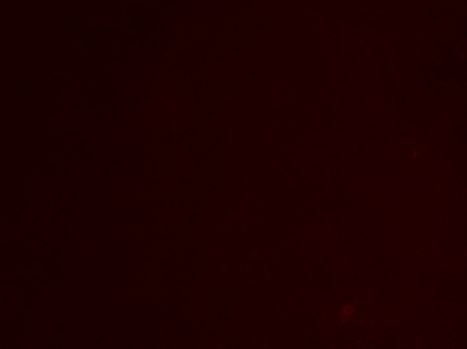

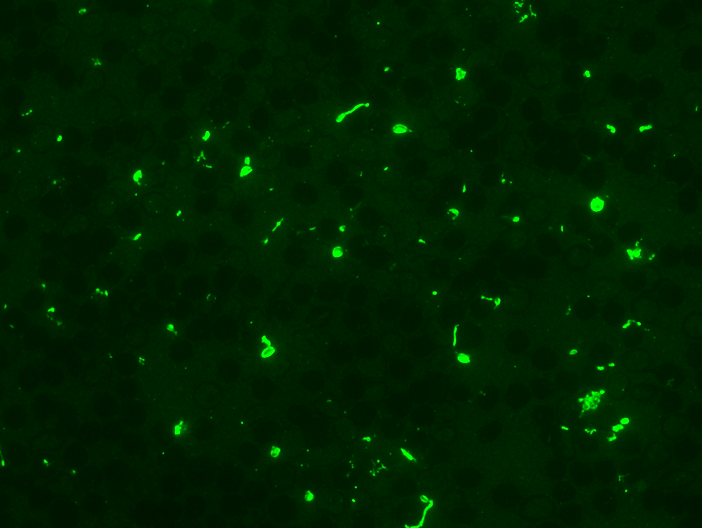

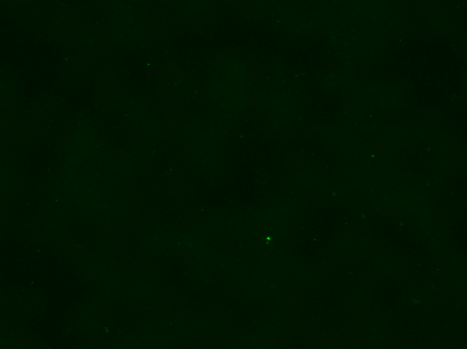

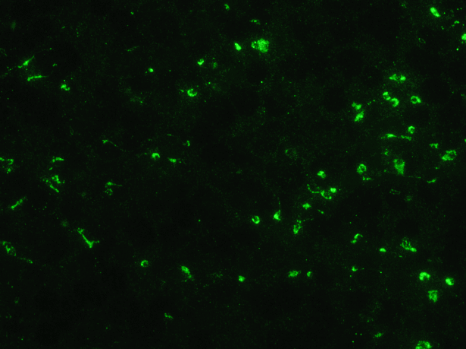

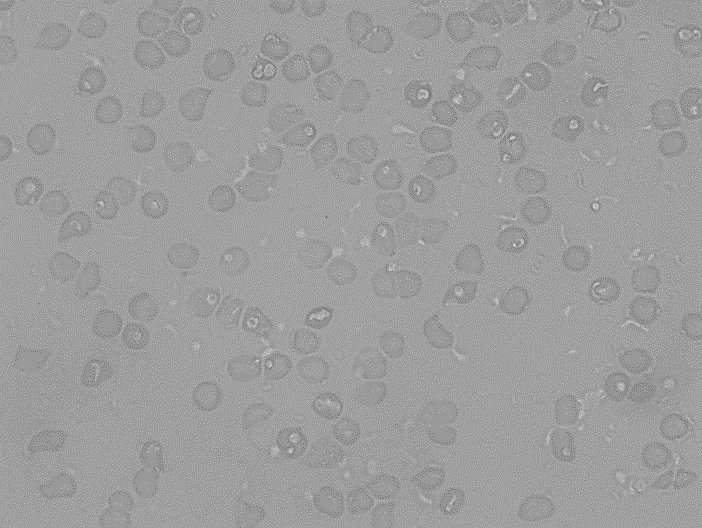

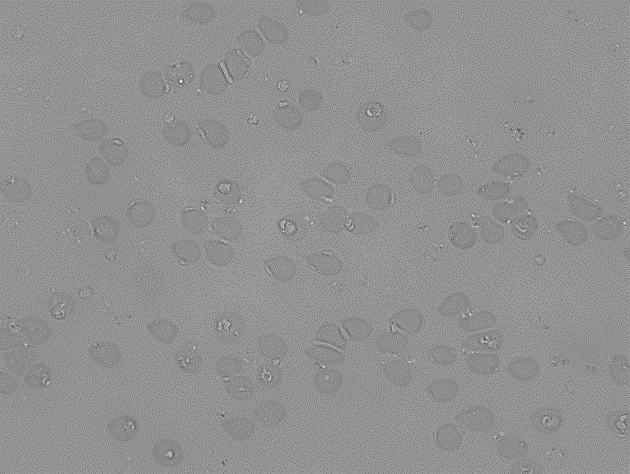


6 dpi, BALB/c

*P. chabaudi*-pRBCs

*B. microti-*pRBCs

**I**

**H**

**G**

**F**

**E**

**D**

**C**

**B**

**A**

*B. microti* anti-serum

Bright field

*P. chabaudi* anti-serum


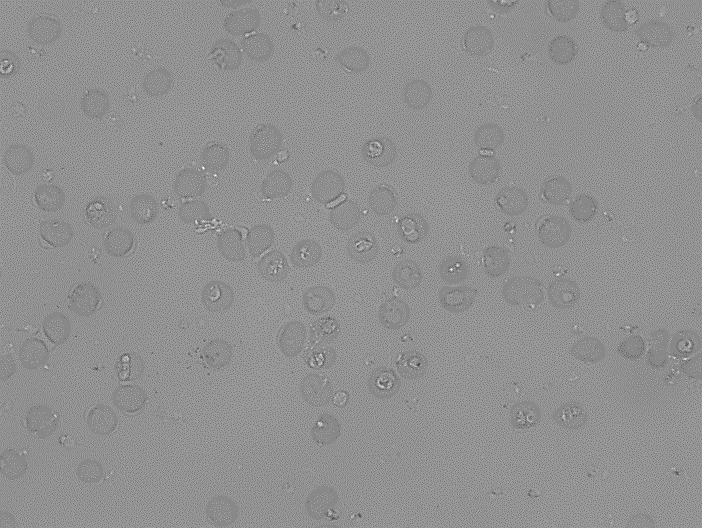

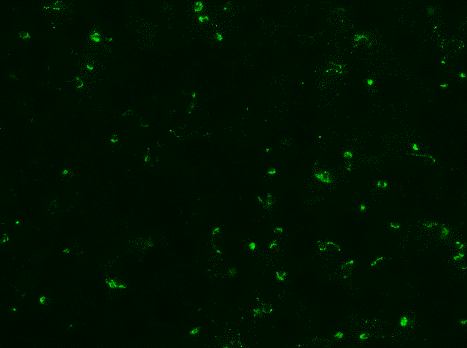

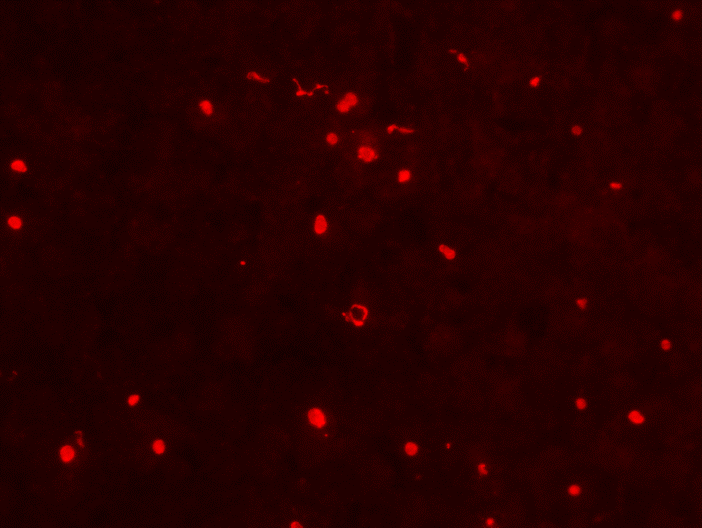


6 dpi, SCID

**L**

**K**

**J**

**Supplementary Figure 2. IFAT for the differentiation of *B. microti-* and *P. chabaudi-*pRBCs at day 6 post *P. chabaudi* challenge infection.** (**A, B, C**): Detection of specificity of *B. microti* anti-serum by using slides coated with *B. microti*-pRBCs and incubated with Alexa-Fluor® 488-conjugated secondary antibody. (**D, E, F**): Detection of specificity of *P. chabaudi* anti-serum by using slides coated with *P. chabaudi*-pRBCs and incubated with Alexa-Fluor® 488-conjugated secondary antibody. Differentiation between *B. microti* and *P. chabaudi* at day 6 post challenge infection with *P. chabaudi* by using slides coated with pRBCs collected from BALB/c mice (**G, H, I**) and SCID mice (**J, K, L**) chronically infected with *B. microti*. Secondary antibody conjugated with Alexa-Fluor® 680 was used against *B. microti* anti-serum, while Alexa-Fluor® 488-conjugated secondary antibody was used against *P. chabaudi* anti-serum.


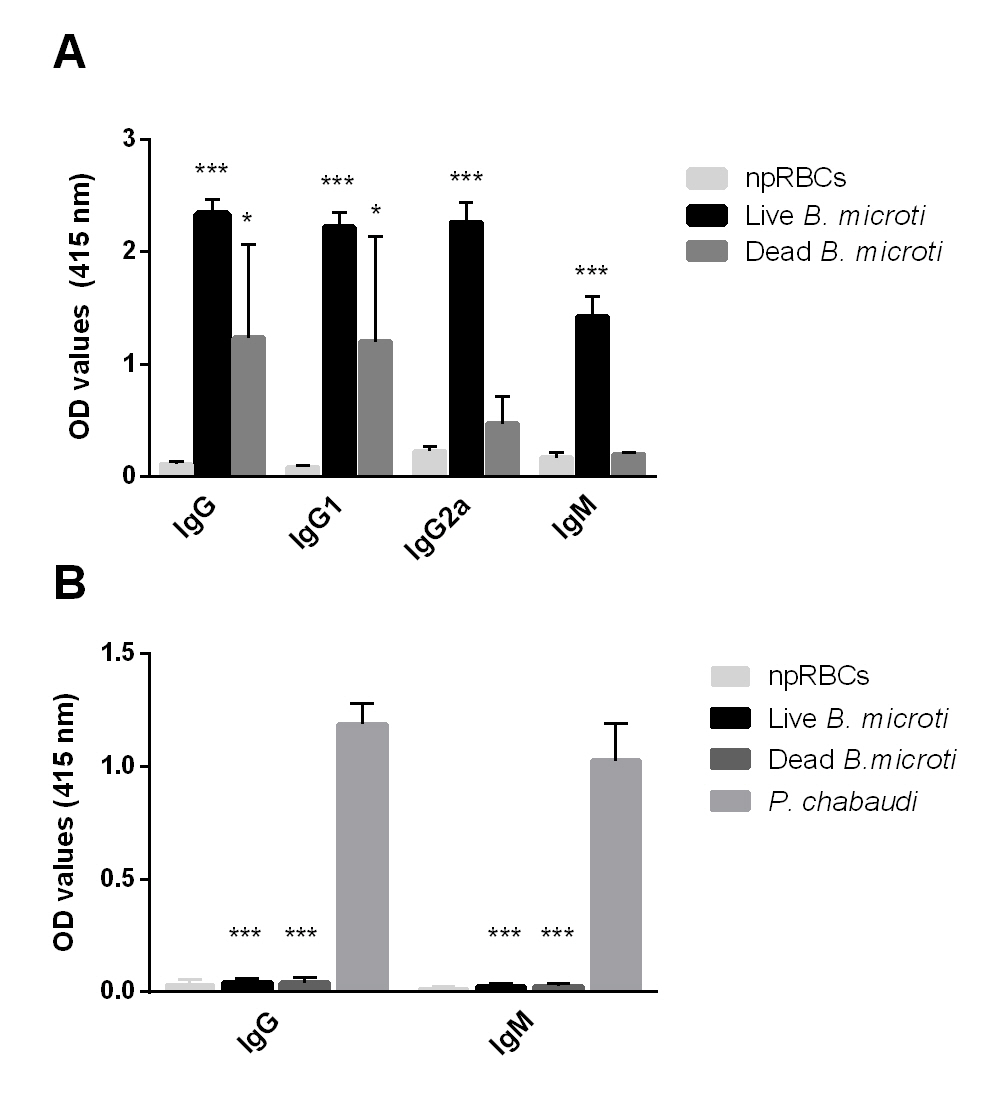


**Supplementary Figure 3**. **Serum antibody levels against *B. microti* and *P. chabaudi* of mice immunized with live and dead *B. microti*.** BALB/c mice were immunized three times at two-week intervals with either glutaraldehyde-fixed *B. microti*-pRBCs (dead *B. microti*), glutaraldehyde-fixed non-parasitized RBCs (npRBCs), or live *B. microti*-pRBCs. The antibody profiles were examined 14 days after the final immunization. (**A**) *B. microti*-specific IgG, IgG1, IgG2a and IgM antibody levels were measured against rBmP32. (**B**) Cross-reactivity of serum IgG and IgM antibodies against *P. chabaudi* crude antigen. Hyper-immune serum obtained from mice challenged with a high dose of *P. chabaudi* was used as a positive control. Asterisks indicate statistically significant differences (*, P < 0.05; **, P < 0.005, and ***, P < 0.0001 compared with control). The results are expressed as mean values ± the SD of five mice.

Clodronate Liposomes

PBS Liposomes

Control

**
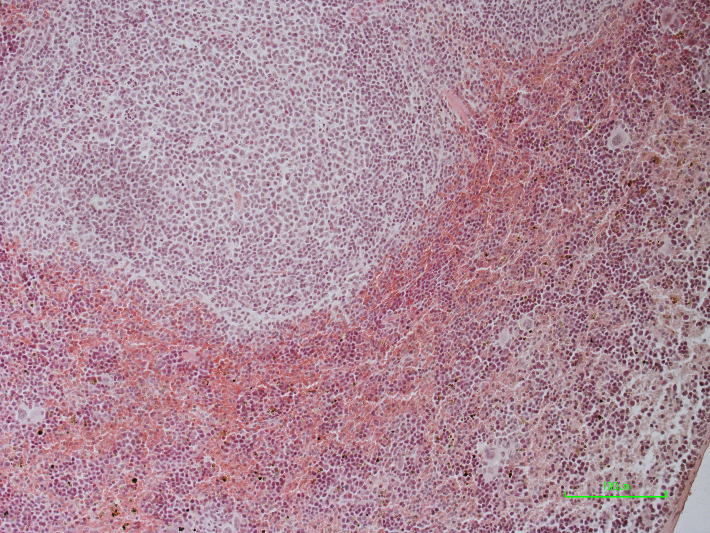

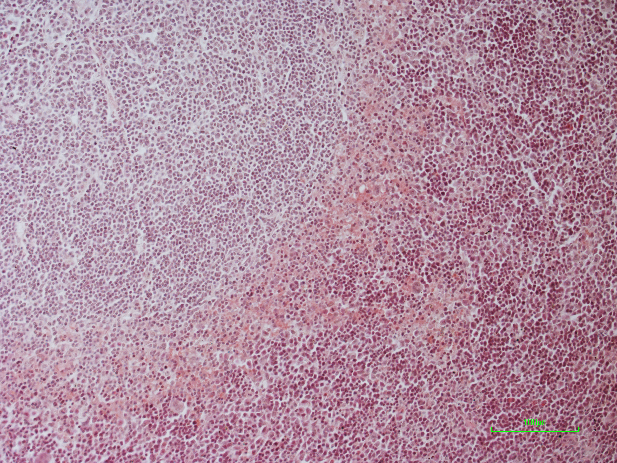

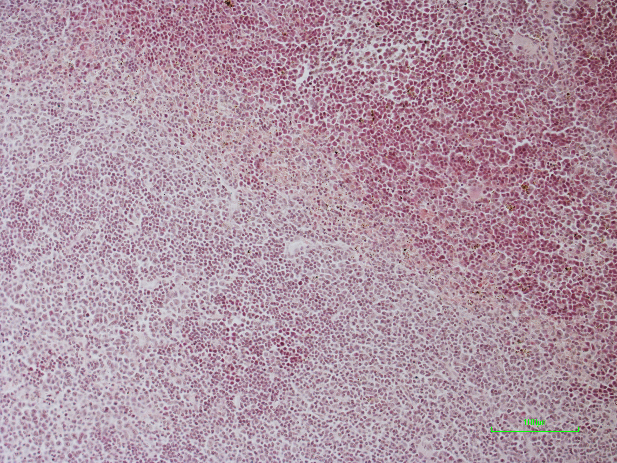
**

**C**

**B**

**A**

**White Pulp**

**F**

**E**

**D**


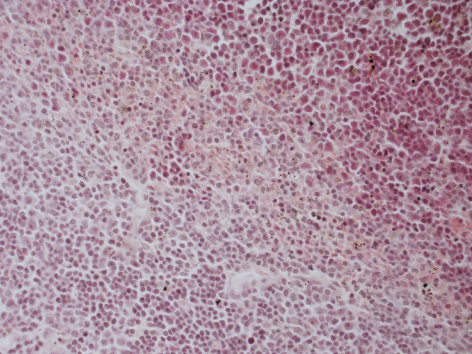


**Marginal Zone**


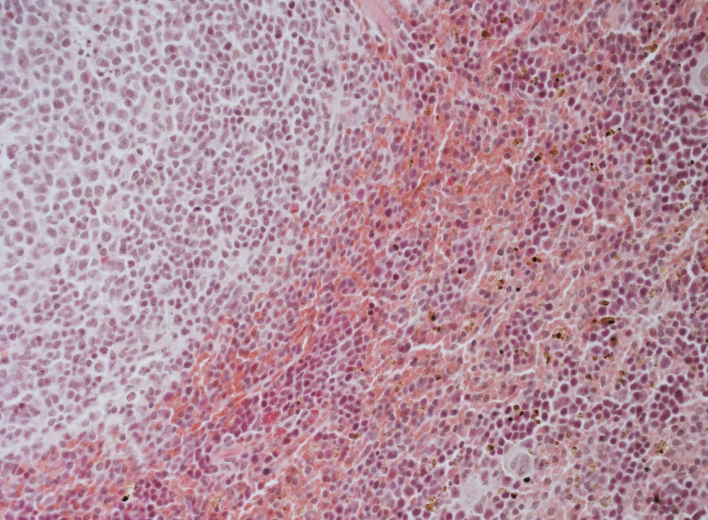


**Marginal Zone**


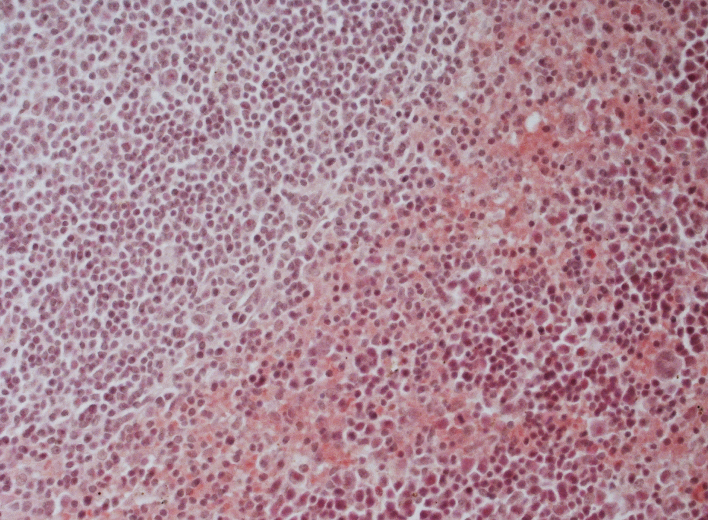


**Marginal Zone**

**White Pulp**

**White Pulp**

**Supplementary Figure 4.** **Confirmation of in *vivo depletion* of macrophages/monocytes in clodronate liposome-treated BALB/c mice.** Mice were primarily infected with *B. microti*, and then challenged with *P. chabaudi* at day 28 post primary infection. Test mice were treated with clodronate liposomes (CLL), mock mice were treated with PBS liposomes (PL), and control mice with sterile PBS. (**A**) Histopathological examination of the spleen: spleen sections stained by Hematoxylin and Eosin; dashed lines enclose the marginal zone, indicating the loss of cells in CLL-treated mice as opposed to mock and control mice. Scale bar = 100 μM.
